# The impact and cost-effectiveness of controlling cholera through the use of oral cholera vaccines in urban Bangladesh: A disease modeling and economic analysis

**DOI:** 10.1371/journal.pntd.0006652

**Published:** 2018-10-09

**Authors:** Ashraful Islam Khan, Ann Levin, Dennis L. Chao, Denise DeRoeck, Dobromir T. Dimitrov, Jahangir A. M. Khan, Muhammad Shariful Islam, Mohammad Ali, Md. Taufiqul Islam, Abdur Razzaque Sarker, John D. Clemens, Firdausi Qadri

**Affiliations:** 1 International Centre for Diarrhoeal Disease Research, Dhaka, Bangladesh; 2 Levin and Morgan, Washington, DC, United States of America; 3 Institute for Disease Modeling Bellevue, WA, United States of America; 4 Independent Consultant, Dennison Street, Waltham, Massachusetts, United States of America; 5 Vaccine and Infectious Disease Division, Fred Hutchinson Cancer Research Center, Seattle, Washington, United States of America; 6 Liverpool School of Tropical Medicine, Liverpool, United Kingdom; 7 Johns Hopkins School of Public Health, Baltimore, Maryland, United States of America; 8 University of Strathclyde, Glasgow, United Kingdom; McGill University, CANADA

## Abstract

**Background:**

Cholera remains an important public health problem in major cities in Bangladesh, especially in slum areas. In response to growing interest among local policymakers to control this disease, this study estimated the impact and cost-effectiveness of preventive cholera vaccination over a ten-year period in a high-risk slum population in Dhaka to inform decisions about the use of oral cholera vaccines as a key tool in reducing cholera risk in such populations.

**Methodology/Principal findings:**

Assuming use of a two-dose killed whole-cell oral cholera vaccine to be produced locally, the number of cholera cases and deaths averted was estimated for three target group options (1–4 year olds, 1–14 year olds, and all persons 1+), using cholera incidence data from Dhaka, estimates of vaccination coverage rates from the literature, and a dynamic model of cholera transmission based on data from Matlab, which incorporates herd effects. Local estimates of vaccination costs minus savings in treatment costs, were used to obtain incremental cost-effectiveness ratios for one- and ten-dose vial sizes. Vaccinating 1–14 year olds every three years, combined with annual routine vaccination of children, would be the most cost-effective strategy, reducing incidence in this population by 45% (assuming 10% annual migration), and costing was $823 (2015 USD) for single dose vials and $591 (2015 USD) for ten-dose vials per disability-adjusted life year (DALY) averted. Vaccinating all ages one year and above would reduce incidence by >90%, but would be 50% less cost-effective ($894–1,234/DALY averted). Limiting vaccination to 1–4 year olds would be the least cost-effective strategy (preventing only 7% of cases and costing $1,276-$1,731/DALY averted), due to the limited herd effects of vaccinating this small population and the lower vaccine efficacy in this age group.

**Conclusions/Significance:**

Providing cholera vaccine to slum populations in Dhaka through periodic vaccination campaigns would significantly reduce cholera incidence and inequities, and be especially cost-effective if all 1–14 year olds are targeted.

## Author summary

While oral cholera vaccines are increasingly being used in the past few years, mainly to curtail or preempt cholera outbreaks, they have yet to be used on a large scale to control endemic cholera in a high-burden country like Bangladesh. This study examines the potential impact on disease and value of vaccinating slum dwellers in Dhaka (and by extension in other cities), who are among those at highest-risk of getting the disease. This analysis suggests that, despite the modest efficacy and limited duration of protection of existing vaccines, mass cholera vaccination can have a significant impact on reducing cholera incidence in the entire population–including those not vaccinated–as a result of herd effects–and can be a cost-effective means of controlling the disease, especially until more long-term measures, such as improved water and sanitation infrastructure, are put in place. These results should assist policymakers and potential donors in determining whether and how to use these vaccines in Bangladesh to control the disease amongst its most vulnerable populations.

## Introduction

The Ganges River Delta and Bay of Bengal, including Bangladesh, are considered the birthplace of cholera and the origin of six of the seven cholera pandemics recorded in modern times[1]. While national population-based estimates of cholera incidence are lacking in Bangladesh, the perception among local experts is that cholera is increasingly becoming an urban disease. Indeed, based on long-term systematic laboratory testing of 2% of all diarrheal patients presenting at the icddr,b (International Centre for Diarrhoeal Disease Research, Bangladesh) hospital in Dhaka (locally known as the “cholera hospital”), this hospital provides care and treatment to approximately 140,000 patients of all ages in each year[2]. Dhaka has also experienced several large cholera outbreaks in the past two decades, especially during widespread floods. During major floods in 2004, 2007 and 2009, icddr,b saw an estimated 30,000 or more cholera cases annually and *V*. *cholerae* overtook rotavirus and ETEC as the main pathogen found among patients with severe diarrhea presenting at the hospital[3]. Several cholera outbreaks have also recently been documented in urban areas in other parts of the country[4, 5].

Residents of slums and low-income districts are especially vulnerable to cholera infection. A systematic sampling of every third diarrheal patient coming to the icddr,b hospital from the low-income area of Mirpur in Dhaka City found *V*. *cholerae* to be the most common pathogen isolated–accounting for 23% of cases, 70% of whom were severely dehydrated [6]. Overcrowded living conditions, inadequate sanitation, and overstressed water systems that are not keeping up with population growth are key contributors to high cholera incidence in urban slums, with tap water supplies often found to be the source. These water supplies, even if initially treated with chlorination, become contaminated during distribution due to illegal connections, leaky pipes and low or negative water pressure–resulting in the mixing of sewerage and drinking water and a dilution in chlorine levels [4, 5]. Contamination of water at the household level, due to poor hygiene, is also common.

The Government of Bangladesh has increasingly expressed interest in controlling cholera since the late 2000s. The Bangladesh delegation to the Executive Board of the World Health Organization (WHO) played a key role in the development and subsequent passage of a resolution by the World Health Assembly in 2011 calling for member states and WHO to strengthen efforts to prevent and control cholera through a series of measures, including the use of oral cholera vaccines (OCVs) “where appropriate, in conjunction with other recommended prevention and control methods and not as a substitute for such methods” [7].

That same year, the Bangladesh Ministry of Health and Family Welfare (MOHFW) played an active role in planning, implementing and monitoring a mass cholera vaccination feasibility study implemented by icddr,b in the Mirpur area of Dhaka, in which more than 123,000 persons one year and above received two doses of the bivalent, whole-cell killed oral cholera vaccine, Shanchol (produced in India), either alone or in combination with the promotion of hand washing and point-of-use safe water treatment interventions [8–10]. The vaccine–administered in two doses at least two weeks apart and licensed for use in persons one year and older–was shown in a clinical trial in Kolkata, India to have an overall efficacy of 65% lasting at least five years [11] and has been used through a global vaccine stockpile to preempt or respond to cholera outbreaks in ten countries from 2013 to October 2016. The Mirpur study found mass cholera vaccination in this impoverished, high-risk area to be feasible–with an overall estimated coverage rate for two doses of 72%, including 67% in adults 18 and older–and generally well accepted by the population [10].

The interest in cholera vaccination among local policymakers has been enhanced by the technology transfer and clinical development of a vaccine identical to Shanchol by a Bangladeshi private sector producer, Incepta Vaccine Ltd. The vaccine, to be marketed as Cholvax, is anticipated to be licensed by the end of 2018 and to cost less than Shanchol (which has a public sector price of $1.85 per dose for single-dose vials).

Analyses of the impact and cost-effectiveness of introducing a new vaccine are increasingly being conducted by countries and donors–most prominently the Gavi Alliance–to inform decisions about implementing or supporting vaccine introductions and which vaccination or targeting strategies to use. Such analyses using the TRIVAC cost-effectiveness model developed by the Pan American Health Organization (PAHO) have reportedly played an important role in decisions by a number of countries in the Latin America and Caribbean region to introduce *Haemophilus influenzae* type b (Hib), pneumococcal conjugate and rotavirus vaccines, as well as more recently in nine countries in Europe, Africa and the Middle East in deciding whether or not to introduce rotavirus or pneumococcal vaccines [12, 13].

Impact and cost-effectiveness analyses can especially be important in the case of a vaccine against a disease like cholera, in which the risk of getting the disease varies greatly by location (due to differing water and sanitation conditions) and by age group. Such analyses can therefore help policymakers make evidence-based decisions on whether or not to use oral cholera vaccines and which geographic areas and age groups to target in order to have the greatest impact for the lowest possible cost.

The purpose of this study was to estimate the impact, cost and cost-effectiveness of preventive cholera vaccination over a ten-year period in a high-risk population of slum dwellers in the city of Dhaka, Bangladesh in order to assist policymakers and global partners in determining whether OCVs should be used in such populations as one of the tools to reduce the cholera risk, and if so, which vaccination strategies would be most effective and efficient. To enhance the relevance and credibility of the results, this study uses local data to model the effectiveness of vaccination on cholera transmission, as well as for several key epidemiological and economic variables.

## Methods

### Ethics statement

This protocol has been approved by the Research Review Committee (RRC) and Ethical Review Committee (ERC) at the icddr,b. All data used for this component was anonymised. Moreover, no active data has been collected for this component of the project.

The assumptions and data used for the major parameters for the analyses are shown in Table 1. Additional parameters for the effectiveness modeling are shown in Table A1 in the technical appendix (S1 Appendix).

**Table 1:** Parameter values used for the impact and cost-effectiveness analyses.

Parameter	Value	Data Source
Proportion of population in targeted thanas living in slums	40%	Gruebner et al. 2014 [15]
Estimated cholera incidence per 1,000 per year in slum areas:		Systematic laboratory surveillance at icddr,b main hospital and satellite hospital in Mirpur for the overall incidence estimate and data from Matlab [18] for age-specific rates (rescaled to average 2.3 (see text)
▪ 0–4 year olds	7.86	
▪ 5–14 year olds	2.65	
▪ Persons 15+	1.38	
▪ All ages (average)	2.3	
Case fatality rate	1.5%	Expert opinion from icddr,b
Duration of cholera illness	4 days	Salomon et al. 2012 [16]
Duration of infection (shedding of *Vibrio cholerae* O1)	5 days	Ali et al. 2011 [17]
Vaccine efficacy over five years (direct protection):		Bhattacharya et al. 2013 [11]
▪ 1–4 year olds	42%	
▪ 5–14 year olds	68%	
▪ Persons 15+	74%	
Cholera vaccination coverage rates:		Average coverage rates from OCV campaigns in Asia, Africa and Haiti in the past 6 years, including Mirpur feasibility study (see S2 Table).
▪ 1–14 year olds	70%	
▪ 15+ year olds	55%	
**Parameters for the economic analysis**		
Direct cost of treating a hospital cholera case in Dhaka	$52.20	Sarker 2016 [23] (see S4 Table)
Vaccine cost per dose:		
▪ Single dose vials	$1.40	Incepta Vaccines Ltd.
▪ 10-dose vials	$0.77	Based on differences in price between one and ten-dose vials of several UNICEF vaccines (see S3 Table)
Vaccine delivery cost per dose:	$0.84 ($1.67 for two dose series)	Sarker et al. 2015 [22]
Vaccine wastage rate:		WHO estimates
▪ Single dose vials	5%	
▪ 10-dose vials	15%	
DALY weight	0.202	Salomon et al 2012 [16]
Discount rate	3%	Fox-Rushby & Hanson 2001 [19]

### Area and population targeted for vaccination

To identify areas in Dhaka at high risk of cholera, we analyzed data from the laboratory surveillance of 2% of hospitalized diarrhea patients who visited the main icddr,b hospital in Dhaka and 10% of patients who came to the icddr,b treatment center in Mirpur from 2011 to 2015. The data include place of residence. Average annual cholera incidence rates by the sub-districts of Dhaka (known as *thanas*) were then estimated, using *thana*-level data from the 2011 census for the denominator. A threshold incidence rate of 1.5 cases per 1,000 per year was used to select high-incidence *thanas*. Fourteen of the city’s 43 *thanas* were found to meet this criteria, with an estimated 2015 population of around 3.5 million (supplemental S1 Table). Not all hospitalized cases of cholera in Dhaka are treated at the two icddr,b facilities, and thus this method may underestimate the number of high-incidence *thanas*, as well as their incidence. However, Mirpur is amongst the areas from where the highest numbers of cholera patients seek treatment at the icddr,b hospitals [6].

To further target those at the highest risk of cholera, the study selected for vaccination slum populations within the 14 high-risk *thanas*, under the assumption that the vast majority of hospitalized cholera cases come from slum areas. The slum population is assumed to make up around 40% of the total population of these *thanas* (or around 1,383,400 people), based on an estimate from the Center for Urban Studies [14].

### Vaccination strategies and target ages modeled in the analyses

The study assumes the use of the bivalent whole-cell oral cholera vaccine to be produced locally (Cholvax). The five-year, age-specific vaccine efficacy rates used for two doses of the vaccine are from the Kolkata clinical trial of Shanchol (42% for 1–4 year olds, 68% for 5–14 year olds, and 74% for persons 15 years and older) [11].

The analysis modeled the impact and cost-effectiveness of mass cholera vaccination campaigns for three increasingly large target age groups: 1–4 year olds, 1–14 year olds, and all persons one year and older. These targeting options were selected based on interviews conducted with MOHFW officials and other stakeholders. Although the vaccine has been shown to provide protection for five years, at least for children over five years and adults, the campaigns–to be conducted in two rounds (one for each dose)–are proposed to take place every three years over the ten-year period of the analysis. This is to account for population mobility in and out of the slum areas–which has the effect of reducing the population’s vaccination coverage over time–as well as the vaccine’s relatively low efficacy rates in the youngest (1–4 year) age group. All three targeting strategies also include the annual vaccination of new birth cohorts through the routine immunization program to protect them during non-campaign years. The first dose can be provided concurrently with the second dose of measles-containing vaccine scheduled at 15 months of age.

We assume a vaccination coverage rate for the two-dose series of 70% for children 1–14 years of age (for both the routine infant vaccination and campaigns) and 55% for persons 15 and above. These estimates are based on an average of coverage rates achieved in several OCV campaigns conducted in different countries in recent years, including the Mirpur feasibility study mentioned above (see S2 Table in supplement).

### Disease parameters and assumptions

A case of cholera is defined in this study as one suffering from acute watery diarrhea requiring a visit to a treatment setting. The estimated average annual incidence rate of cholera requiring treatment in the target slum population is 2.3 per 1,000. This rate was derived by applying the proportion of diarrheal cases that were found to be confirmed cholera cases through the systematic testing of patients at the icddr,b Dhaka hospital and Mirpur treatment center to the total number of patients seeking care at the hospital for severe diarrhea[6]. Although the incidence of reported cholera varies by thana, we assume that all high-risk populations in Dhaka have the same high cholera incidence rate and therefore use the same rate for the entire target population. Under the assumption that nearly all cholera cases coming to the icddr,b Dhaka hospital are from slum areas, the denominator was the estimated size of the slum population in Dhaka, based on government population data and the Center for Urban Studies estimate of the percentage of Dhaka residents who live in slums (40%) [14]. Age-specific incidence rates were derived by applying the age distribution of cholera cases found through on-going laboratory-supported cholera surveillance in Matlab from 1997 to 2001 to the overall incidence of 2.3/1,000 per year. The estimated incidence rates are 7.86 per 1,000 for children under five years of age, 2.65/1,000 for 5–14 year olds, and 1.38/1,000 for persons fifteen and older. Thus, using these estimates, pre-school children are 5.7 times more susceptible and school-aged children nearly twice as susceptible of becoming infected with cholera requiring treatment as adults in this population. This increased susceptibility of children might actually reflect increased exposure to cholera due to age-related behavioral differences, but the actual biological mechanism behind this does not affect the model.

In the absence of data on cholera case fatality rates, an estimated rate of 1.5% was used, based on the opinion of experts at icddr,b. Estimates of the average duration of illness and duration of infection come from the literature [15, 16].

### Mathematical modeling of cholera transmission and impact of vaccination

To estimate the impact of different vaccination targeting strategies on the incidence of cholera in this population over a ten-year period, we used a mathematical model that simulates the dynamics of cholera transmission. This model is based on a previously-published model of cholera transmission in Matlab that used times-series data of cholera incidence and other epidemiological data from Matlab from 1997 to 2001 [17]. Details on the model, including all parameters and differential equations used, are given in the technical appendix (S1 Appendix).

**Fig 1:**
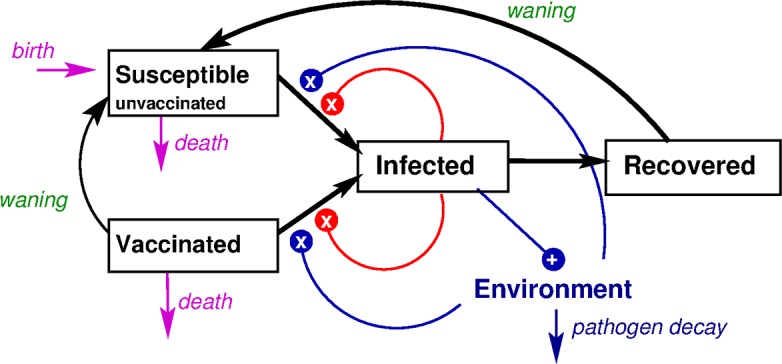
Simplified diagram of the mathematical model of cholera transmission in Bangladesh. Boxes represent individuals, who can be susceptible (vaccinated or unvaccinated), infected, or recovered from cholera, and arrows between the boxes are possible transitions people make between these states. Infected individuals shed Vibrio into the environment. Susceptible individuals transition to infected at a rate governed by both the number of infected individuals and the amount of Vibrio in the environment, represented by lines ending in Xs.

The model simulates how a person can be infected by another individual–either symptomatic or asymptomatic–or from the environment (e.g., via water) (Fig 1). It also simulates the effect of immunity on disease transmission from having been infected (natural immunity) or having been vaccinated. In brief, the model places people in one of four compartments: 1) susceptible to cholera, 2) infected and symptomatic, 3) infected but asymptomatic, and 4) recovered and immune. The concentration of *V*. *cholerae* in the environment (water) is tracked in another compartment. Ordinary differential equations are used to model the transition of people between compartments over time, which is affected by such factors as the number of infected persons in the community, the level of *V*. *cholerae* in the environment, the time it takes for an infected individual to clear the infection, and the efficacy of either natural or vaccine-induced immunity over time. The model was calibrated to simulate the seasonality of cholera in Matlab over a one-year period.

The model assumes four different levels of susceptibility to infection based on age groups (children under two years of age, 2–4 year olds, 5–14 year olds, and adults 15 and older). It also assumes that vaccine efficacy is based on the age at vaccination (1–4, 5–14 and 15+ years old), using the age-specific vaccine efficacy estimates from Bhattacharya et al. 2013 described above [11].

In the dynamic model of cholera transmission, the rate of cholera infection is proportional to the number of infected individuals and the amount of *Vibrio* in the environment. Therefore, vaccination not only reduces the number of cases among vaccinees, but even non-vaccinated individuals are protected indirectly because the averted cases among vaccinees reduce everyone’s risk of infection. The dynamic model was used to estimate the magnitude of this effect. Thus, the indirect (herd) protective effects of cholera vaccination are built into this dynamic model. The incorporation of herd effects, along with the simulation of the seasonal pattern of the disease, are meant to provide a more accurate picture of the impact of cholera vaccination on disease incidence over time than would a static outcomes model, in which vaccination does not reduce the incidence of cholera in the unvaccinated population so there are no herd effects.

In adjusting the model from Matlab to Dhaka, a migration factor was added to account for the high mobility of slum populations in cities like Dhaka. The model thus replaces a portion of each vaccinated population group with non-vaccinated individuals each year at a constant rate and assumes that these non-vaccinated newcomers have the same level of cholera susceptibility as the baseline (pre-vaccination) population. This has the effect of reducing vaccination coverage in the target population over time. Since little data on migration in and out of the slums of Dhaka are available, we modeled three annual migration rates: 0%, 10% and 25%, and used 10% as the base case for the main analyses. However, we assumed that the size of the at-risk population is fixed over the 10-year period of the analysis (i.e., as many people leave as enter the targeted areas).

The output of the model is the number of symptomatic cases of cholera per year, including those not treated, for each age group and each vaccination targeting strategy, varied by migration rates. We then translated the results to adjust for the age structure in Dhaka (which is somewhat different than that in Matlab), based on census data. We assume that most cholera illness is either mild or otherwise unreported, so we computed a "reporting rate" that, when multiplied by the age-adjusted number of symptomatic cases derived from 100 stochastic runs of the model using an unvaccinated population, produced 2.3 reported cases per 1,000 population per year (the estimated annual incidence of cholera requiring treatment in the target population, as described above). The analysis of cases averted is based on the "reported" number of cases produced by the model.

For each vaccination strategy modeled, we obtained the number of cases prevented each year and cumulatively over ten years by subtracting the number of cases predicted once vaccination is implemented from the expected number of cholera cases if no vaccination takes place. The number of deaths averted was calculated by multiplying the number of cases prevented by the assumed case fatality rate of 1.5%.

### Cost-effectiveness analyses

Measures of cost-effectiveness were obtained by dividing the net cost of vaccination over the ten-year period of the analysis by the cumulative number of cases, deaths and disability adjusted life years (DALYs) prevented as a result of vaccination for each of the targeting strategies. The net vaccination cost is the cost of the vaccination program minus the estimated savings in treatment costs resulting from a reduction in cholera incidence due to vaccination. The resulting incremental cost-effectiveness ratios (ICERs)–cost per case averted, cost per death averted, and cost per DALY averted–were calculated for two different vaccine presentations: single-dose vials and ten-dose vials.

As is standard, DALYs averted were calculated using DALY weights, a standard discount weight, and life expectancies using methods described in a paper by Fox-Rushby and Hanson [18]. No age weights were used in the analysis.

This economic analysis takes a health provider perspective, as opposed to a societal perspective. The vaccination costs are assumed to be paid by the public sector (government and/or donors) and only the cost of treating cholera paid by the health care provider are included. Thus, the costs of cholera illness borne by individuals, such as out-of-pocket expenses for medicines, transport and lodging for caregivers and the indirect costs of loss wages of patients or their caregivers from missing work–are not included in the treatment cost estimates. Nor were any private costs related to vaccination, such as the cost of transportation or of missing work to get vaccinated. The cost-of-illness from a societal perspective would include these private costs–resulting in greater treatment savings–and thus our cost-effectiveness measures will be slightly more conservative than if a societal perspective was used.

Cost-effectiveness thresholds based on a country’s per capita gross domestic product (GDP) have often been used as a measure of the cost-effectiveness of a health intervention, with a cost per DALY averted that is equal to or less than the GDP per capita indicating that the intervention is “very cost-effective” [19]. However, these thresholds have been criticized as too limited as the sole or even a major determinant in decision-making, especially since they do not take into account a country’s specific context, including its ability to afford the intervention [20]. Therefore, in addition in comparing the cost per DALY averted results to per capita GDP, we also examine the affordability of cholera vaccination, in terms of cost per vaccinees and program cost as a percentage of the routine EPI budget. A univariate deterministic sensitivity analysis was conducted to show which variables have the greatest impact on cost-effectiveness. In the Excel spreadsheet that calculated cost-effectiveness, we designated distribution functions for the variables with uncertainty and then ran the Monte Carlo simulations. The gamma distribution function was used to estimate three variables: unit vaccine cost, vaccine delivery cost, and cholera treatment cost, while the beta distribution function was used to estimate two variables that had values that were between 0 and 1: the case fatality rate and cholera incidence. This analysis varies the values of key input parameters–case fatality rate, cholera incidence rates, vaccine price, cost of treatment, and vaccine delivery cost–one by one to estimate how these affect the outcome. Monte Carlo simulations were conducted multiple times (10,000) by drawing random values from the distribution functions for the input parameters using Ersatz software (version 1.3). Two distribution functions are used to model uncertainty: 1) beta for incidence and case fatality rates, variables with values between 0 and 1; and 2) gamma for vaccine, delivery, and treatment costs. For the gamma distribution, the parameters were a shape parameter α and a mean parameter *β*.

For the beta distribution, the parameters are two positive shape parameters, denoted by *α* and *β*, that appear as exponents of the random variable and control the shape of the distribution.

The input variable that is the most influential on cost per DALY averted is the one with the longest confidence interval, as will be shown in the tornado graph.

### Key parameters used for the economic analyses

#### Cost of vaccination

The analysis assumes that each person will receive two doses of the locally-produced Incepta vaccine. The estimated price for single-dose vials sold to the public sector is US $1.40, based on a discussion with the producer. The estimated price per dose for ten-dose vials is $0.77, or 45% less than the single-dose vials, based on an average differential in UNICEF prices per dose between single- and ten-dose vials for several childhood vaccines (S3 Table).

The projected operational costs of delivering the vaccine were taken from a study of oral cholera vaccination costs conducted during the OCV feasibility study in Mirpur, Dhaka [21]. These costs included: 1) start-up costs: training materials, social mobilization, and training; and 2) service delivery costs: personnel salaries, supplies, vaccine transport, cold chain storage and equipment and waste management. The vaccine delivery cost was estimated at $0.84 per dose or $1.67 per person for the two-dose series. The total cost of cholera vaccination, including the vaccine price and delivery cost for two doses, is therefore $4.47 per person for single-dose vials and $3.21 for ten-dose vials, before vaccine wastage rates are taken into account.

To obtain the number of doses to be used each year and over the ten-year period for each vaccination strategy, we estimated the number of persons to be vaccinated in each targeted age group by multiplying the target population by the assumed age-specific coverage rates (70% for 1–14 year olds and 55% for persons 15+). Vaccine wastage rates of 5% for one-dose vials and 15% for ten-dose vials were then added to estimate the total number of doses required, based on WHO estimates.

#### Cost of cholera illness

Data on the cost of treating cholera patients in Dhaka were derived from an analysis conducted at the icddr,b hospital (S4 Table). The analysis used a micro-costing, bottom-up approach that calculated the hospital’s total costs of treating hospitalized cholera cases in the year 2013. These include direct medical costs for cholera treatment (e.g., medical staff, medical supplies, equipment costs), direct non-medical costs (e.g., for non-medical staff, meals), and costs shared among different wards of the hospitals for such services as pharmacy, administration, hospital management information system and laundry. The proportion of shared costs incurred by cholera patients was estimated using the percent of cholera patients among all patients treated at the hospital, as estimated from the 2% systematic surveillance system. The total costs to the hospital for treating cholera for the year were then divided by the estimated number of cholera patients seen that year. The average cost of treating a cholera patient came to around $52.20. This average cost for a hospitalized case is in the range of costs estimated in the burden of disease studies conducted by the Diseases of the Most Impoverished (DOMI) program [22]–from $31.50 in Matlab (based on surveillance data from 1998–2003), $35.40 in Kolkata, India (2003–2005), $47.20 in Beira, Mozambique (2004), and $205.70 in Jakarta, Indonesia (2001–2003).

## Results

### The predicted impact of cholera vaccination on disease incidence

**Fig 2:**
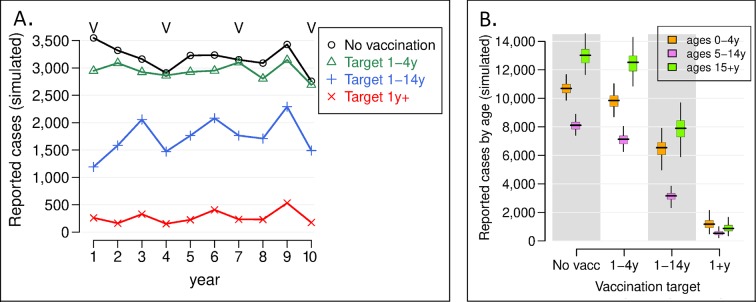
Predicted number of reported cholera cases in the targeted population (n = 1.4 million) following implementation of the vaccination program. A) The simulated number of reported cases. Different target populations were targeted for vaccination in the model, and the average of 100 stochastic runs per strategy is plotted. The Vs above the lines indicate the years when vaccination campaigns occurred. B) The number of reported cases by age group when different age groups are targeted for vaccination. The boxes indicate the inter-quartile interval of 100 model runs, the horizontal lines the median result, and the vertical lines the 95% observed interval. Variation in model runs are a result of the stochastic nature of the model, not parameter uncertainty. *V indicates years OCV campaigns would take place.

**Fig 3:**
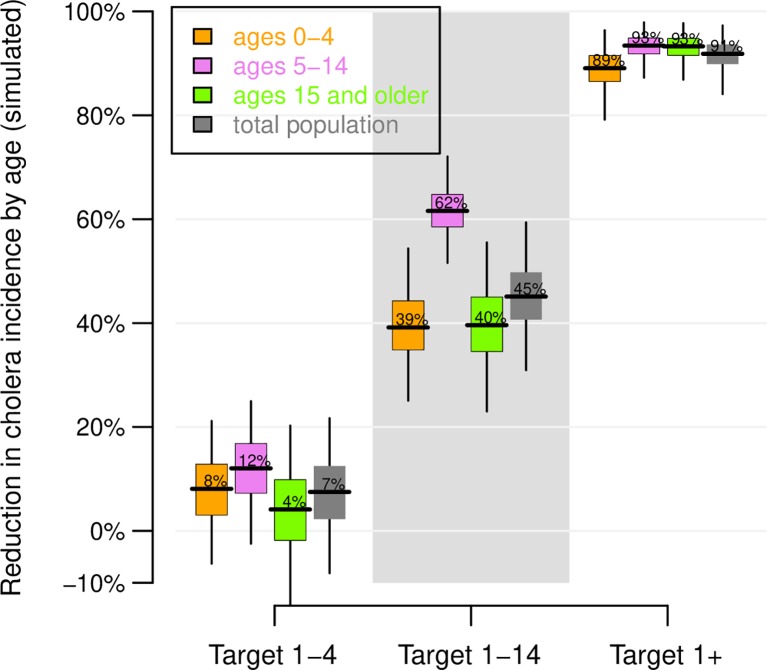
Predicted effectiveness of vaccination. The percent reduction in cholera incidence by age group over 10 years with respect to simulations with no vaccination are plotted. The distribution of effectiveness estimates is computed by taking 10,000 random draws with replacement from 100 stochastic runs with vaccination and 100 stochastic runs with no vaccination. The boxes indicate the inter-quartile interval of 100 model runs, the horizontal lines the median result, and the vertical lines the 95% observed interval. Variation in model runs are a result of the stochastic nature of the model, not parameter uncertainty.

**Fig 4:**
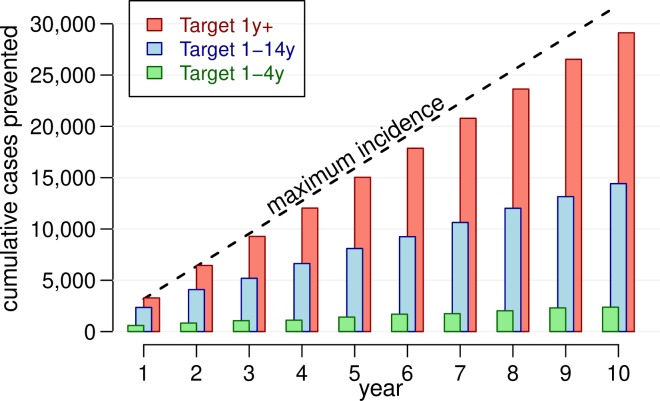
Cumulative number of cholera cases prevented in the targeted population, by vaccination strategy. The average results from 100 stochastic runs per vaccination scenario are plotted. The dashed line shows the cumulative population incidence of cholera of 2.3 cases per 1000 per year.

Using a base case of 10% annual migration (i.e., the target population is replaced at a rate of 10% a year), Fig 2A shows the predicted number of reported cholera cases each year in the target population of around 1.4 million people once the vaccination program–consisting of mass vaccination campaigns every three years and annual vaccination of the new birth cohort–is implemented, for each vaccination strategy. 2B depicts the total number of cases for the ten-year period by vaccination strategy and age group. The model was at equilibrium when interventions were simulated, so the simulations with no vaccination also reflect the incidence of cholera before vaccination. The percent reduction in incidence for each vaccination strategy is shown in Fig 3, while the cumulative number of cases prevented over the ten-year period is shown in Fig 4. Results showing 95% confidence intervals are presented in S1 Appendix.

If no vaccination or other cholera intervention program is enacted, there would continue to be, on average, around 3,200 cases of cholera in the study population presenting at health facilities each year, or more than 32,000 cases over the ten-year period of the analysis. A strategy of vaccinating only children 1–4 years of age would reduce cholera incidence in the overall targeted population by around 7% − preventing around 2,411 cases over ten years or 241 cases per year on average (Figs 3 and 4 and Table 2). Expanding the target vaccination group to 1–14 year olds would prevent around 14,400 cases over ten years, reducing incidence in the overall population by 45%. Vaccinating all ages one and above would prevent more than 29,100 cases over this period, reducing the overall cholera burden in the target population by 91%. Viewed from another perspective, for every reported case prevented in the overall population of nearly 1.4 million, 123 children 1–4 years of age would need to be vaccinated compared to 80 children 1–14 years old and 102 persons one year and above (Table 2). The strategy of targeting 1–14 year olds is thus the most efficient.

**Table 2:** Ten-year outcome measures used for the cost-effectiveness analyses, assuming a 10% annual migration rate.

Vaccination strategy (age group targeted)	Number persons vaccinated	Number of cases averted	Number of deaths averted	Number of DALYs averted	Number persons vaccinated per case averted
1–4 years	296,142	2,411	36	1,043	123
1–14 years	1,152,984	14,430	216	6,241	80
1+ years	2,976,785	29,114	437	10,366	102

The herd effects of cholera vaccination are clearly shown in these results. The reduction in cases overall and in each age group is modest when vaccination is limited to 1–4 year olds, who make up around 7% of the total study population. However, expanding vaccination to all 1–14 year olds not only reduces the number of cases amongst these children, it also reduces incidence in adults (who are not vaccinated) by 40% (Fig 3). Thus, while 1–14 year olds account for around 30% of the population, vaccinating them would cut cholera incidence in the entire population by 45%. When adults are also vaccinated, more than 90% of cholera cases would be prevented.

We tested these results with different levels of annual population migration, which dilutes vaccination coverage of the population over time. When the entire population one year and above is targeted for vaccination and there is no migration, cholera transmission virtually stops and the overall effectiveness of the program is nearly 100% (Figure S3 in S1 Appendix). We also simulated vaccination campaigns every 5 years instead of every 3 years. In these scenarios, we assumed that vaccine protects individuals for 5 years. Vaccinating every 5 years is somewhat less effective than vaccinating every 3 years when 10% annual migration is assumed (Figure S5A and S5B in S1 Appendix), and becomes less effective when the migration rate is high (Figures S5C and S5D). If annual migration is 25%, the effectiveness of this strategy may fall below 90%, while when migration is 10% a year, effectiveness is above 90%. The effectiveness of vaccinating 1–14 year olds on the overall cholera incidence in the population is reduced from nearly 48% at 0% migration to 45% at 10% migration and to 41% at a 25% migration rate. When only 1–4 year olds are vaccinated, the effectiveness is around 7%, regardless of the migration level.

### Cost of vaccination

The projected costs of vaccination for the ten-year period by vaccination strategy and vial size are shown in Table 3. Including annual vaccination of infants in all scenarios, the total costs are estimated at $1.5 - $1.9 million (depending on the vial size) for the strategy targeting 1–4 year olds, $4.4 - $5.9 million for the strategy targeting 1–14 year olds, and $10.8 - $14.3 million if all persons one year and older are targeted. The annual costs over the ten-year period therefore range from approximately $145,600 to $1.08 million if ten-dose vials are used and from $193,080 to $1.4 million if single-dose vials are used. The cost per vaccine recipient, including vaccine wastage, is estimated at $4.62 for single-dose vials and $3.48 for ten-dose vials.

**Table 3:** Vaccination costs by targeting strategy and vial size for 3 vaccination campaigns coupled with annual infant vaccination over 10 years, US$.

Vaccination strategy (age group targeted)	Population to be vaccinated* (a)	No. doses (b)	Vaccine costs ** (c)	Vaccine delivery cost (d)	Total cost (c+d)	Total costs with annual infant vaccination 12–15 month olds	Total annual cost	Cost per vaccinee
**Single-dose vials**								
12–15 mo. (routine vaccination)	122,017	256,236	$359,630	$203,769	$563,398			$4.62
1–4 years	296,142	621,899	$872,840	$494,558	$1,367,398	$1,930,796	$193,080	
1–14 years	1,152,984	2,421,267	$3,398,269	$1,925,484	$5,323,753	$5,887,151	$588,715	
1+ years	2,976,785	6,251,248	$8,773,681	$4,971,230	$13,744.911	$14,308,310	$1,430,831	
**Ten-dose vials**								
12–15 months (routine vaccination)	122,017	256,236	$221,066	$203,769	$424,835			$3.48
1–4 years	296,142	621,899	$536,540	$494,557	$1,031,097	$1,455,933	$145,593	
1–14 years	1,152,984	2,421,267	$2,088,936	$1,925,484	$4,014,420	$4,439,244	$443,924	
1+ years	2,976,785	6,251,248	$5,393,233	$4,971,230	$10,364,464	$10,789,299	$1,078,930	

* Assumes 70% coverage for children 1–14 and 55% for adults 15+

** Includes vaccine wastage.

Table 4 shows the net cost of vaccination once the savings in treatment costs resulting from the program are subtracted from the total vaccination program costs. The estimated savings in treatment costs for the ten-year period range from around $126,000, if the program is limited to 1–4 year olds, to $1.5 million if all persons one year and above are included.

**Table 4:** Total and net costs of OCV vaccination by strategy and vial size over ten years.

Targeted age group	Total cost of vaccination (a)	Cost of illness per case (b)	Number of cases averted (c)	Treatment savings (d) (b x c)	Net cost of vaccination (e) (a-d)
**Single-dose vials**					
1–4 years	$1,930,796	$52.20	2,411	$125,839	$1,804,957
1–14 years	$5,887,151		14,430	$753,229	$5,133,922
1+ years	$14,308,310		29,114	$1,519,770	$12,788,539
**Ten-dose vials**					
1–4 years	$1,455,933	$52.20	2,411	$125,839	$1,330,094
1–14 years	$4,439,255		14,430	$753,229	$3,686,025
1+ years	$10,789,299		29,114	$1,519,770	$9,269,528

Note: All strategies include routine annual vaccination of children aged 12–15 months.

### Incremental cost-effectiveness ratios

The results of dividing the net vaccination costs by cases, deaths, and DALYs averted for each vaccination strategy and vaccine vial size are shown in Table 5. The option of vaccinating 1–14 year olds would be by far the most cost-effective, with a cost per case averted of $255 - $356 and a cost per DALY averted of between $591 and $823. The next most cost-effective strategy would be vaccinating all ages one year and above, with a cost per case averted of $318 - $439 and cost of DALY averted of $894 - $1,234–1.5 times higher than the 1–14 year old targeting strategy. On the other hand, limiting cholera vaccination to 1–4 year olds would cost $1,276 - $1,731 per DALY averted–making it 2.2 times less cost-effective than the strategy of targeting 1–14 year olds and 1.4 times less cost-effective than the option of vaccinating all persons one year and above.

**Table 5:** Cost-effectiveness ratios for cholera vaccination by vaccination strategy and vial size, US$, over ten years.

Vaccination Strategy (targeted age group)	Cost per case averted	Cost per death averted	Cost per DALY averted
**Single-dose vials**			
1–4 years	$749	$49,915	$1,731
1–14 years	$356	$23,719	$823
1+ years	$439	$29,283	$1,234
**Ten-dose vials**			
1–4 years	$552	$39,783	$1,276
1–14 years	$255	$17,030	$591
1+ years	$318	$21226	$894

**Fig 5:**
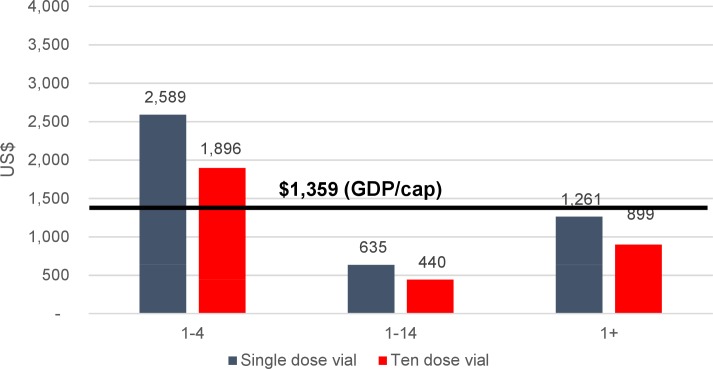
Cost per DALY averted from cholera vaccination against the GDP per capita in Bangladesh, by program strategy, US$.

Comparing the cost per DALY averted to the GDP per capita as a measure of cost-effectiveness, all vaccination strategies would cost less per DALY averted than the country’s per capita GDP threshold ($1,359) [23], except the option of vaccinating 1–4 year olds only using single-dose vials, indicating that they would be cost-effective using this definition (Fig 5). However, the 1–4 year old vaccination strategy using ten-dose vials, as well as the strategy of vaccinating all ages one and above using single-dose vials barely fall below the threshold.

### Sensitivity analysis

**Fig 6:**
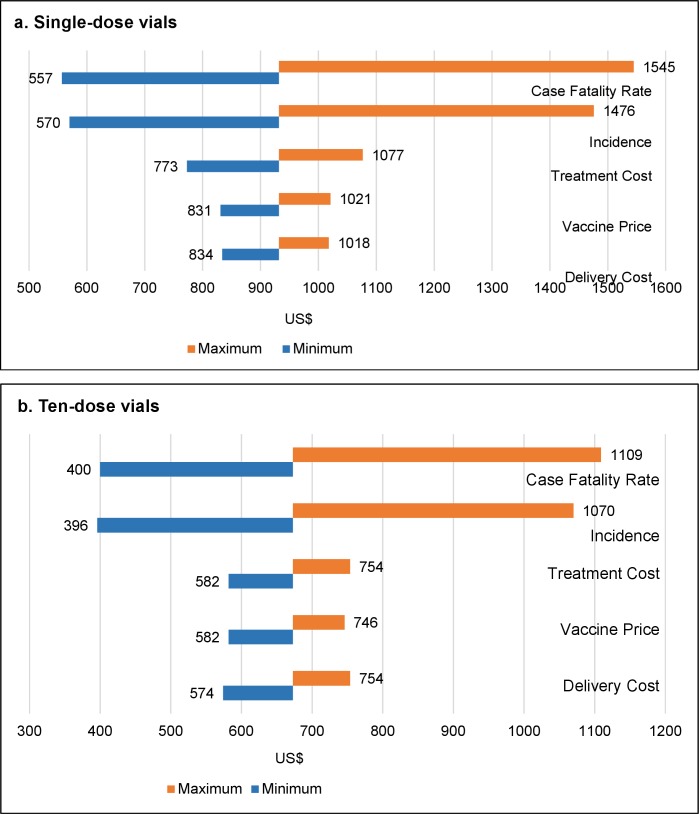
Incremental cost-effectiveness ratios (cost per DALY averted) for vaccination of 1–14 year olds with parameters varied, by vial size (US$): a) single dose vial; b) ten dose vial.

Univariate deterministic sensitivity analyses were conducted on variables with the greatest uncertainty in their values: case fatality rate (CFR), vaccine price, delivery cost, treatment cost and cholera incidence. The cost per DALY averted estimates vary most with changes in CFR, cholera incidence rates, and cost of treatment, while varying vaccine price and delivery costs has less effect on incremental cost-effectiveness ratios (Table 6 and Fig 6). However, even when any of the five parameters are varied, the strategy targeting 1–14 year old children for vaccination is the most cost-effective, regardless of the vaccine vial size, followed by vaccination of all persons one year and older.

**Table 6:** Incremental cost-effective ratios (cost per DALY averted) for vaccination of children 1–14 years and rank of influence when key variables are varied, by vial size (US$).

Variable	Type of Distribution and Parameters	Minimum	Maximum	Rank of influence
**Single dose vials**				
Case fatality rate	Beta, α1 = 15; α2 = 985	$557	$1,545	1
Incidence	Beta, α1 = 23, α2 = 977	$570	$1,476	2
Treatment cost	Gamma, α = 6.82; β = 136	$773	$1,077	3
Vaccine price	Gamma α = 16; β = 5.6	$831	$1,021	4
Vaccine delivery cost	Gamma, α = 70; β = 7	$834	$1,018	5
**Ten dose vials**				
Case fatality rate	Beta, α1 = 15; α2 = 985	$400	$1,109	1
Incidence	Beta, α1 = 23, α2 = 977	$396	$1,070	2
Treatment cost	Gamma, α = 6.82; β = 136	$557	$771	3
Vaccine delivery cost	Gamma, α = 70; β = 7	$574	$754	4
Vaccine price	Gamma, α = 16; β = 5.6	$582	$746	5

Table 7 shows the mean value and 95% confidence interval for the cost per DALY averted by age group and vial size when the uncertain parameters are varied in the Ersatz Monte Carlo simulations. The results again show that the lowest cost per DALY averted is found when the age group 1–14 is vaccinated using either vial size.

**Table 7:** Mean and median incremental cost-effectiveness ratios (cost per DALY averted) with parameters varied, by age group and vial size (US$).

Vaccination age group	Cost per DALY averted (median)	Cost per DALY averted (mean)	95% confidence interval	Standard deviation
**Single-dose vials**				
Children 1–4	$1,798	$1,949	$876 - $3,889	776
Children 1–14	$852	$925	$395 - $1,858	380
Persons 1+	$1,278	$1,389	$602 - $2,802	602
**Ten dose vials**				
Children 1–4	$1,329	$1,420	$680 - $2,705	529
Children 1–14	$615	$664	$288 - $1,323	269
Persons 1+	$937	$1,005	$452 - $1,960	394

### Affordability

Affordability and the budgetary impact of cholera vaccination is another critical factor in assessing the value for money of this intervention. The cost per vaccinee (vaccine and service delivery) in this analysis is $3.48 if ten-dose vials are used and $4.62 if single-dose vials are used–regardless of the age group targeted for vaccination (Table 3). The annual cost of vaccination in the proposed areas of Dhaka for the most cost-effective strategy targeting 1–14 year olds − $443,924 if ten-dose vials are used, and $588,715 if single-dose vials are used–represents 0.15% to 0.2% of the 2018 routine EPI budget of nearly $300 million [24].

## Discussion

This analysis predicts that a program in which all children 1–14 years of age are targeted in mass cholera vaccination campaigns every three years, coupled with annual vaccination of infants 12–15 months old through the routine immunization program, would reduce cholera incidence by 45% over 10 years in a population of around 1.4 million slum dwellers in high-risk areas of Dhaka, Bangladesh, preventing 14,400 cases, including in many adults, and cost $4–6 million over ten years. This would be by far the most cost-effective of the three program options, as well as the most efficient, in terms of the number of vaccinations per case averted.

Vaccinating all ages one and above would, on the other hand, reduce cholera incidence in this population by more than 90% over 10 years, preventing more than 29,000 reported cases. However, this strategy would be considerably less cost-effective than the one targeting 1–14 year olds, and would cost, on average, $1.1–1.4 million per year or $11–14 million over ten years. The option of vaccinating only young children (1–4 year olds) would have minimum impact on the overall cholera incidence (7% reduction) and despite its lower costs ($1.5 - $1.9 million), it would be the least cost-effective of the three strategies analyzed. Moreover, if we target 1–14 years age group a total of 14,430 cases will be averted whereas 2,411 cases will be averted if we target 1–4 years age group. However, these very young children, who are most vulnerable to severe cholera, would be included in the middle option targeting 1–14 year olds.

The results for the 1–14 year old and all-ages strategies show the power of herd effects from cholera vaccination, given that the analysis assumes that only a portion of the targeted population would receive the vaccine (70% of children and 55% of adults) and since direct protection from the vaccine is quite modest (ranging from 42% in children under five to 68% in 5–14 year olds over five years). Our results are in general agreement with observations of herd immunity observed in large trials [8, 25]. A large-scale cluster randomized trial of cholera vaccination was implemented in Dhaka, and effectiveness was modest (37% over 2 years) [8]. The effectiveness could have been reduced by both the high migration rate and contamination between vaccinated and unvaccinated clusters. The large-scale vaccination campaigns represented by our modeling results are likely to result in higher effectiveness than cluster-randomized trials.

These findings reinforce those of past cost-effectiveness analyses of cholera vaccination conducted in Bangladesh and elsewhere, which also found that a strategy of vaccinating children was more cost-effective than a strategy that included vaccinating adults–a key factor being the much higher incidence rates of endemic cholera in children than in adults [24, 26, 27]. However, the results also show that limiting vaccination to children under five–those typically targeted by national immunization programs and mass vaccination campaigns–would have a minimal impact on cholera incidence and would be considerably less cost-effective than the other two targeting strategies. Key reasons are the lower efficacy of the vaccine in this youngest age group (42%), and the low level of herd protection from vaccinating such a small proportion (≈7%) of the general population. Although the efficacy of OCV among children under 5 years old has been found to be about half that of older children in endemic settings in several studies [28–30], the our model could underestimate the impact of vaccinating young children if they contribute more to transmission than older children and adults.

A strength of this study is the identification of high-risk areas in Dhaka that would be strong candidates for a targeted cholera vaccination program in Dhaka. The study also improves upon previous cost-effectiveness analyses by using local data on the cost of recently-conducted vaccination campaigns.

However, a number of limitations of the analysis need to be pointed out. The mathematical model was calibrated to the dynamics of cholera transmission in the rural area of Matlab. The transmission dynamics and epidemiological patterns of cholera (e.g., survival of *V*. *cholerae* in the environment, seasonal patterns, age distribution of cases, ratio of asymptomatic to symptomatic infections) may differ somewhat in urban slums, such as those in Dhaka. Although the population of Matlab and Dhaka differ, we believe that the attack rates of cholera in these highly vulnerable populations subject to seasonal monsoon-driven epidemics are similar. Uncertainty around these parameters, as well as those governing the dynamics of *V*. *cholerae* in the environment, is difficult to resolve and can skew projections of outbreak sizes [31]. However, the mathematical model of cholera transmission was used solely to estimate the magnitude of indirect protection from mass vaccination, which should be robust to variation in these parameters as long as the disease attack rates are calibrated. In addition, in the absence of population-based cholera incidence data, an incidence estimate of treated cases (2.3/1,000) was extrapolated from data from two hospitals run by icddr,b. While the majority of cholera cases requiring care in Dhaka are reportedly treated at these two facilities, the omission of cases treated elsewhere would have the effect of under-estimating the true cholera incidence rate, making our analyses conservative. On the other hand, the estimated incidence rate is based on the assumption that all cases coming to these hospitals are from the estimated population who live in slums, which may not be the case. There are also uncertainties with the cholera case fatality rate and level of migration in Dhaka. The uncertainty of these key parameters (CFR, incidence and migration rates) was addressed in the sensitivity analyses, which in general show that the results, when comparing the different targeting strategies, remained similar when the values of these parameters were varied.

Concerning the economic analyses, the average cost of treating hospitalized cases of cholera in Dhaka was taken from a cost study at the main icddr,b hospital, which may have higher costs of treatment (and a higher standard of care) than in other health facilities in the city. In addition, the same cost estimate was applied to all cases, regardless of age, while the cost of treating children may differ somewhat from that of treating adults, as found in past studies of the cost of cholera illness [22, 32]. However, the sensitivity analysis showed that even varying the cost of treatment estimates by a factor of two in either direction did not substantially change the incremental cost-effectiveness ratios.

It should also be noted that this analysis focuses on cholera vaccination in a highly-targeted population of around 1.4 million people considered at highest risk of cholera, in a city of 8.5 million, with more than 18 million in the Greater Dhaka Area [33]. Since there are many slums–and cases of cholera–beyond those in the 14 *thanas* included in this analysis, controlling or even eliminating cholera in Dhaka would likely require expanding the target population to other areas in (and possibly surrounding) the city. This would increase the costs of the program and could also reduce its cost-effectiveness, if the cholera incidence rates in other areas are lower than the rate used in this analysis.

The producer of Cholvax has recently indicated that the public sector price for single-dose vials could be reduced to as low as $1.10 (vs. $1.40 assumed in the base analysis). This lower price would significantly reduce the costs and thus increase the cost-effectiveness of cholera vaccination under the modeled scenarios.

Cost-effectiveness is not the only factor that policymakers and donors need to consider when making decisions about whether or not to implement cholera vaccination in high-risk areas and which age groups to target. Another key consideration is affordability, as the financial realities in Bangladesh make it unlikely that the country can readily implement all potentially cost-effective interventions. The most cost-effective strategy–that of vaccinating 1–14 year olds in this population of around 1.4 million people–would cost, on average, ≈$444,000 - $589,000 per year, while the most expansive strategy of targeting all ages one and above would cost around 60% more ($1.1–1.4 million). The 1–14 year old option would thus add 0.15%– 0.2% to the annual national immunization program budget (of around $300 million), while the all-ages strategy would add 0.4%-0.6%. Assuming vaccine protects for 5 years for all age groups, we found that spacing the campaigns to every 5 years led to more cases 4 or 5 years after campaigns but only decreased average effectiveness over 10 years only a modest amount. However, the 5-year campaigns were less effective when migration was higher. If protection from vaccination wanes more rapidly among young children [30], then less frequent vaccination campaigns might not adequately protect them. However, expanding the vaccination program to other areas of Dhaka or to other cities in Bangladesh would increase these costs. In terms of the estimated cost per vaccinee − $4.62 for single-dose vials and 3.48 for ten-dose vials–this compares to a cost estimate for rotavirus vaccination (using Rotarix) of $5.46 - $5.98 per child vaccinated from an analysis conducted by PATH (Clint Pecenka, personal communications).

If protection from vaccination wanes more rapidly among young children, then less frequent vaccination campaigns might not be effective. One way to reduce the costs of cholera vaccination, and thus increase its affordability and cost-effectiveness, would be to decrease the frequency of the vaccination campaigns from every three years to every five years, given that the vaccine has been shown to provide protection for at least five years, at least among persons five years and older. However, in a highly mobile area such as the slums of Dhaka, population migration would erode coverage and decrease the effectiveness of vaccination over time. If one assumes, for example, an annual migration rate of 10%, 50% of the population will have been replaced with unvaccinated people within five years, greatly reducing the level of protection in this population and potentially leading to outbreaks three or four years after a campaign. Conducting vaccination campaigns every five years may be an appropriate strategy in a less mobile population.

Government policymakers in Bangladesh have expressed interest in providing cholera vaccination in combination with other interventions to reduce cholera and water-borne diseases, as recommended by WHO [34]. A comprehensive cholera control program that combines vaccination with improvements in water distribution and water quality–such as by increasing the number of legal water connections and placing water pipes far from sewer systems—should, in fact, create synergies that result in a more rapid reduction in disease than any intervention on its own [35]. Cholera vaccination could also be a means of accelerating control of the disease before adequate water and sanitation improvements can reach the most vulnerable populations. In addition, cholera vaccination could be provided during periodic intensive routine immunization (PIRI) activities or other vaccination campaigns (e.g., measles-rubella), which would further reduce its costs.

Another key factor to consider in deciding whether or not to introduce a new vaccine is fairness and equity [20]. Because cholera predominantly strikes the most impoverished and marginalized populations, who are also those with the least access to quality health care services, cholera vaccination, especially using a strategy targeting high-risk areas, would reduce these inequities.

While cholera incidence rate estimates are not available for other urban areas of Bangladesh, it is likely that in cities where the disease is known to be endemic or where outbreaks have occurred in the recent past and where slum conditions are similar to those in Dhaka, the risk of cholera will be similar to that found in the slums of Dhaka. Thus, it is reasonable to assume that the results of this analysis can be generalized to slum populations in other cholera-affected urban areas of the country. The findings from icddr,b’s prospective cholera surveillance currently underway in twenty-two mainly urban sites throughout the country will help increase our understanding of the cholera burden in other parts of the country and thus determine the relevance of the findings of our analysis to other urban areas. This strategy of targeting urban slums for special disease control efforts would also align with a new national government priority of improving health in slums in Bangladesh.

## Supporting information

S1 Table Selected thanas of Dhaka for the analysis and their populations. (DOC)

S2 Table Coverage rate estimates from past OCV campaigns. (DOC)

S3 Table UNICEF prices for one- and ten-dose vials of several EPI vaccines. (DOC)

S4 Table Estimated average cost of treating a hospitalized cholera patient at the icddr,b hospital, 2013 (US$). (DOC)

S1 Appendix Mathematical modeling methodology and additional results. (DOC)
