# Supplementary material for: The impact and cost-effectiveness of controlling cholera through the use of oral cholera vaccines in urban Bangladesh: A disease modeling and economic analysis
Source: PLoS Negl Trop Dis. 2018 Oct 9;12(10):e0006652. doi: 10.1371/journal.pntd.0006652 (PMC6177119; doi:10.1371/journal.pntd.0006652)
Supplement: S1 Table — (DOC) [file pntd.0006652.s001.doc]

**S1 Table. Selected *thanas* of Dhaka** for the analysis and their populations

| **Thana** | **Annual Incidence per 1,000** | **Census population in 2011** | **Estimated population in 2015*** | **Estimated slum population (40%) in 2015** |
| --- | --- | --- | --- | --- |
| Biman Bandar | 8.47 | 10,626 | 11,229 | 4,492 |
| Tejgaon | 4.11 | 148,255 | 156,671 | 62,668 |
| Kotwali | 3.54 | 62,087 | 65,612 | 26,245 |
| Jatrabari | 3.20 | 184,575 | 195,053 | 78,021 |
| Gulshan | 2.25 | 253,050 | 267,416 | 106,966 |
| Kafrul | 2.74 | 396,182 | 418,673 | 167,469 |
| Mirpur | 2.14 | 500,373 | 528,779 | 211,512 |
| Mohammadpur | 2.14 | 355,843 | 376,044 | 150,418 |
| Dakshinkhan | 2.11 | 255,931 | 270,460 | 108,184 |
| Ramna | 1.94 | 200,973 | 212,382 | 84,953 |
| Shah Ali | 1.82 | 115,489 | 122,045 | 48,818 |
| Kamrangir Char | 1.71 | 93,601 | 98,915 | 39,566 |
| Badda | 1.66 | 536,621 | 567,085 | 226,834 |
| Darus Salam | 1.51 | 159,139 | 168,173 | 67,269 |
| **Total** | **2.22** | **3,272,745** | **3,458,538** | **1,383,416** |
