# Supplementary material for: The impact and cost-effectiveness of controlling cholera through the use of oral cholera vaccines in urban Bangladesh: A disease modeling and economic analysis
Source: PLoS Negl Trop Dis. 2018 Oct 9;12(10):e0006652. doi: 10.1371/journal.pntd.0006652 (PMC6177119; doi:10.1371/journal.pntd.0006652)
Supplement: S2 Table — (DOC) [file pntd.0006652.s002.doc]

S2 Table. Coverage rate estimates from past OCV campaigns

| **Campaign** | **Coverage with 2 doses** | | **Context/comments** | **Source** |
| --- | --- | --- | --- | --- |
|  | **Children** | **Adults** |  |  |
| Ethiopia 2015 | 81% overall (no breakdown by age group) | |  |  |
| Haiti 2013 (Petite Anse and Cerca Carvajal) | 1-4 yr: 68-82%  5-14 yr: 78-84% | 56-71% | Range is for 2 locations | Tohme et al. 2015 |
| Guinea 2012 | ≈82-86% | ≈65-70% | Reactive vaccination to outbreaks, so motivated population | Luquero et al. 2013 |
| Orissa 2011 | 1-17: 60% | 40% | Endemic area | Kar 2014 |
| Mirpur, Dhaka 2011 demonstration | 1-17: 80% | 66.7% | Demonstration, so intensive social mobilization | Khan 2013 |
| Zanzibar 2010 (using Dukoral) | 1-4y=60%  5-14y=66% | 39% | 50% overall coverage | Khatib et al. 2012 |
| **Average (not weighted)** | **73.4%** | **55.4%** |  |  |

Sources:

Tohme RA, Francois J, Wannemuehler K, Iyengar P, Dismer A, Adrien P et al. Oral cholera vaccine coverage, barriers to vaccination, and adverse events following vaccination, Haiti, 2013. Emerging Infectious Diseases 2015/June, 21 (6):98Emerging Infectious Diseases 2015/June, 21 (6):984-991 .

Lugeroa FJ, Grout L, Ciglenecki I, Sakoba K, Traore B, Heile M et al. First outbreak response using an oral cholera vaccine in Africa: vaccine coverage, acceptability and surveillance of adverse events, Guinea, 2012. PLoS Neglected Tropical Diseases 2013/October; 7(10):e24665.

Kar SK, Sah B, Patnaik B, Kim YH, Kerketta AS, Shin S et al. Mass vaccination with a new, less expensive oral cholera vaccine using public health infrastructure in India: the Odisha model. PLoS Neglected Tropical Diseases 2014/February; 8(2):e2629.

Khan IA, Saha A, Chowdhury F, Khan AI, Uddin MJ, Begum YA et al. Coverage and cost of a large oral cholera vaccination program in a high-risk cholera endemic urban population in Dhaka, Bangladesh. Vaccine 2013; 31:6058-64.

Khatib AM, Ali M, von Seidlein L, Kim DR, Hashimn R, Reyburn R et al. Effectiveness of an oral cholera vaccine in Zanzibar: findings from a mass vaccination campaign and observational cohort study. Lancet Infectious Diseases 2012/Nov; 12(11):837-44.
