# Supplementary material for: The impact and cost-effectiveness of controlling cholera through the use of oral cholera vaccines in urban Bangladesh: A disease modeling and economic analysis
Source: PLoS Negl Trop Dis. 2018 Oct 9;12(10):e0006652. doi: 10.1371/journal.pntd.0006652 (PMC6177119; doi:10.1371/journal.pntd.0006652)
Supplement: S3 Table — (DOC) [file pntd.0006652.s003.doc]

S3 Table. UNICEF prices for one- and ten-dose vials of several EPI vaccines

| **Vaccine** | **One-dose Price** | **Ten-dose Price** | **% Reduction** |
| --- | --- | --- | --- |
| MMR | 2.37 | 1.13 | 52% |
| Pentavalent | 2.25-2.35 | 1.15-1.94 | 34% |
| Hepatitis B | 0.42 | 0.20 | 52% |
| **Average Reduction** |  |  | **45%** |
