# Supplementary material for: The impact and cost-effectiveness of controlling cholera through the use of oral cholera vaccines in urban Bangladesh: A disease modeling and economic analysis
Source: PLoS Negl Trop Dis. 2018 Oct 9;12(10):e0006652. doi: 10.1371/journal.pntd.0006652 (PMC6177119; doi:10.1371/journal.pntd.0006652)
Supplement: S4 Table — (DOC) [file pntd.0006652.s004.doc]

**S4 Table. Estimated average cost of treating a hospitalized cholera patient at the icddr,b hospital, 2013 (US$)**

| **Variable** | **Estimate** |
| --- | --- |
| Number of hospitalized patients 2013 | 121,970 |
| Cholera confirmed cases (estimated) | 19,515 |
| Total cost of cholera treatment | US$ 10,19,401 |
| Average length of stay in short-stay unit (days) | 1.01 |
| Average length of stay in long-stay unit (days) | 1.47 |
| Proportion of recurrent costs | 71.97% |
| Proportion of shared costs | 14.98% |
| Proportion of capital costs | 13.04% |
| Treatment cost per patient | US$ 52.23 |
