# Supplementary material for: The impact and cost-effectiveness of controlling cholera through the use of oral cholera vaccines in urban Bangladesh: A disease modeling and economic analysis
Source: PLoS Negl Trop Dis. 2018 Oct 9;12(10):e0006652. doi: 10.1371/journal.pntd.0006652 (PMC6177119; doi:10.1371/journal.pntd.0006652)
Supplement: S1 Appendix — (DOC) [file pntd.0006652.s005.doc]

### S1 Appendix. Mathematical modeling methodology and additional results

## 1. Model of cholera transmission in Matlab, Bangladesh

The mathematical model of cholera transmission is based on the one described in Dimitrov et al 2014 and was implemented in Matlab R2012a (The MathWorks, Inc.) and is available at https://github.com/FredHutch/Cholera-Model-Bangladesh. The model was calibrated to the dynamics of cholera and demographics of Matlab, Bangladesh, and we translated model results to a Dhaka population as described in section A.3. The changes made to the model did not affect the dynamics of cholera in an unvaccinated population with no migration, so the model calibration and parameters except for those involving vaccine efficacy are exactly as described in Dimitrov et al 2014.

The model aggregates the population in compartments by disease status and age. The disease compartments are unvaccinated susceptible (S), vaccinated susceptible (V), symptomatically infected (I), asymptomatically infected (A), or recovered and immune (R) from cholera (Figure S1). Vaccinated individuals are additionally stratified into 3 groups by their age at the time of vaccination because we assumed differential vaccine efficacy based on age at vaccination, which diverges from the original model described in Dimitrov et al 2014. Infected individuals recover after five days on average and are immune to infection until they transition back to the susceptible state at a rate of 1/3 per year. The concentration of *V. cholerae* in the environment (water) is tracked in an additional compartment (W). Susceptible individuals may become infected by direct contact with infected individuals or by exposure to *V. cholerae* in the environment.

The model divided the population into four age cohorts: children under 2 years old, pre-school aged children (2 to 4 years old), school-aged children (5 to 14 years old), and adults (15 years old and older). Younger age groups are more susceptible to infection. Births are modeled by adding unvaccinated susceptibles to the youngest age cohort each year, and deaths are modeled by removing individuals from all age cohorts. Birth and age-specific mortality rates were based on data from the Matlab Health and Demographic Surveillance System. Cohorts are aged by moving individuals into the next older age compartment at the appropriate rates.

Cholera can be transmitted from infected individuals or through contamination in the water compartment (W). Twenty percent (*p)* of the infections are symptomatic, 10% of which seek treatment (*r*, the reporting rate). The asymptomatically infected individuals (proportion *1-p* of all infections) are less infectious and shed bacteria into the environment at a lower rate than those symptomatically infected.

A migration process was added to the model. Migration replaces a fraction of the population at a constant rate with unvaccinated individuals. We replace a fraction of each vaccinated population compartment by a sufficient number of unvaccinated people distributed proportionally by the size of the compartments. Migration rates of up to 25% are explored in the Results. This migration mechanism mimics population replacement from an infinitely large endemic source that have the same monthly prevalence of cholera as the baseline population. This has the effect of reducing vaccine coverage at a constant rate.

Vaccinated individuals in the model are less likely to become infected upon exposure than unvaccinated individuals of the same age. Protection depends on the age at the time of vaccination, with young children (1-4 years old) being infected at a rate 42% lower than unvaccinated children of the same age, school-aged children (5-14 year-old) being infected 68% less than unvaccinated children their age, and adults 74% less likely (Bhattacharya et al 2013). Because vaccine efficacy is associated with the age at which an individual is vaccinated, vaccine protection does not change when a vaccinee ages out into an older age cohort. In the previous model, it was assumed that vaccine efficacy was associated with the age at time of exposure, so a vaccinee's protection could change as he or she aged into age cohorts that has a different efficacy. Vaccination is modeled by including three “vaccinated” compartments per age group, each associated with one of the age-specific efficacies tied to the time of vaccination. For example, a vaccinated six-year-old would have different protection depending on whether the vaccination occurred before the age of 5 years (42% protection) or after (68% protection). Vaccinated individuals in the model remain protected for three years with no waning of protection. We assumed that upon infection, vaccinated individuals have the same probability of becoming symptomatic as and are as infectious as non-vaccinated individuals.


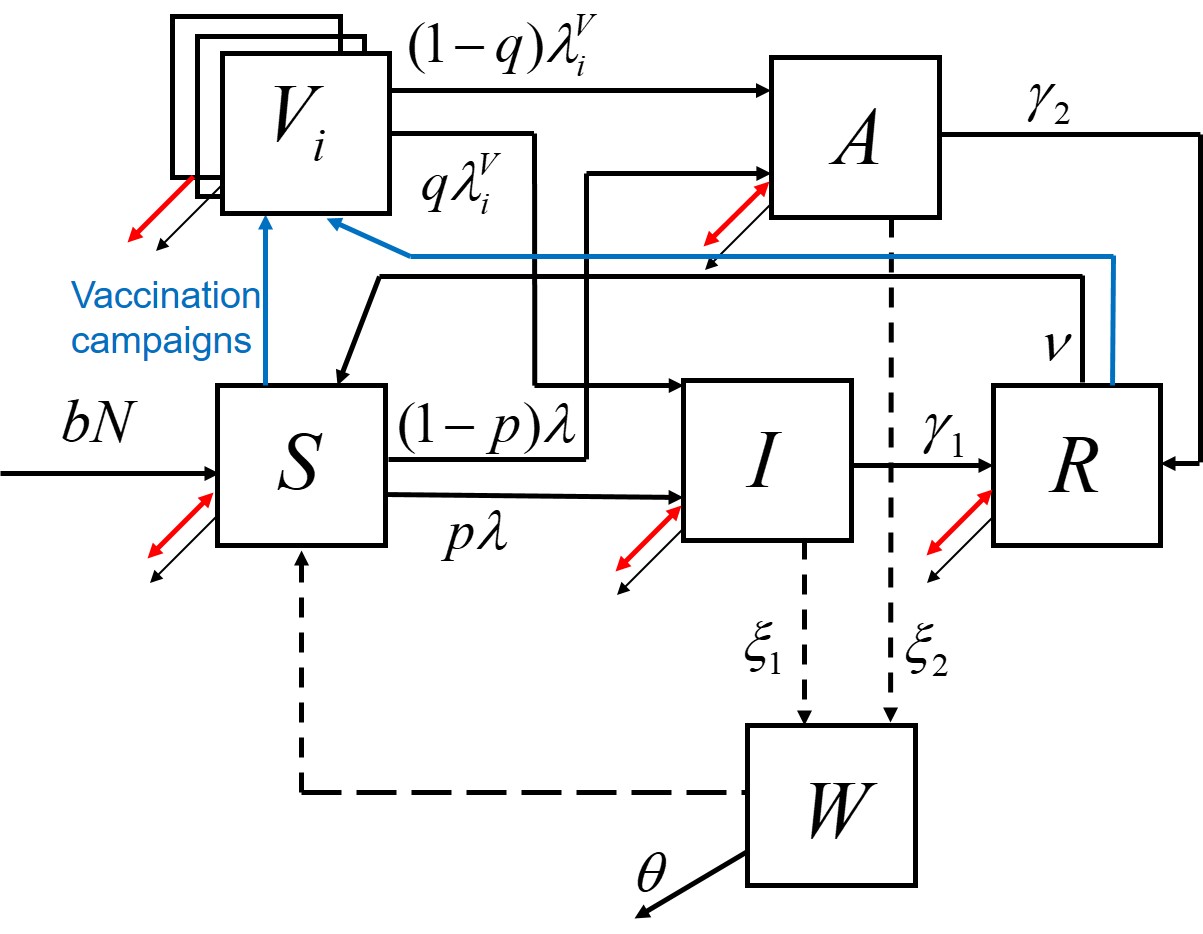


**Figure S1. Flow diagram of the mathematical model of cholera transmission.** Individuals are aggregated in compartments by cholera status as susceptible unvaccinated (S), susceptible vaccinated (V), cholera cases (I), asymptomatically infected (A), and recovered (R). Vaccinated individuals are additionally stratified in 3 groups (i=1,2,3) by their age at the time of vaccination. The concentration of *Vibrio cholerae* in the environment is tracked in a separate compartment (W). Proportion *p* of the susceptibles and proportion *q* of the vaccinated who become infected are symptomatic (cholera cases), and a fraction *r* of these are reported and used to fit the model to surveillance data. Infected individuals shed bacteria into the environment and cause indirect transmission of Cholera (dashed lines). Red arrows represent the flows due to migration. The blue arrows represent the transfer of individuals during vaccination campaigns. The simulated population is additionally stratified by age (not shown).

The epidemic dynamics between vaccination campaigns are described by a set of differential equations for each age group (i=1 for infants age 0-1 years, i=2 for preschoolers age 2-4 years, i=3 for school children 5-14 years, and i=4 for adults age 15+ years). Separate vaccinated compartment by the age at vaccination (j=1 for those vaccinated at ages 1-4 years, j=2 for those vaccinated at ages 5-14 years, j=3 for those vaccinated at age 15 years and older):

$$\begin{matrix} \frac{dS_{i}}{dt}=b_{i}N+md_{i}^{s}N_{i}+a_{i-1}S_{i-1}+\nu R_{i}-(\lambda_{i}^{0}+\mu_{i}+m+a_{i})S_{i} \\ \frac{dV_{i}^{j}}{dt}=a_{i-1}V_{i-1}^{j}-(\lambda{}_{i}^{j}+\mu_{i}+m+a_{i})V_{i}^{j}, j=\text{1,2,3} \\ \frac{dI_{i}}{dt}=md_{i}^{i}N_{i}+p\lambda_{i}^{0}S_{i}+q\sum_{j=1..3} \lambda{}_{i}^{j}V_{i}^{j}+a_{i-1}I_{i-1}-(\mu_{i}+\gamma_{1}+m+a_{i})I_{i} \\ \frac{dA_{i}}{dt}=md_{i}^{a}N_{i}+(1-p)\lambda_{i}^{0}S_{i}+(1-q)\sum_{j=1..3} \lambda{}_{i}^{j}V_{i}^{j_{i}}+a_{i-1}A_{i-1}-(\mu_{i}+\gamma_{2}+m+a_{i})A_{i} \\ \frac{dR_{i}}{dt}=md_{i}^{r}N_{i}+\gamma_{1}I_{i}+\gamma_{2}A_{i}+a_{i-1}R_{i-1}-(\mu_{i}+\nu+m+a_{i})R_{i} \\ \frac{dW}{dt}=\xi_{1}\sum_{i=1}^{4} I_{i}+\xi_{2}\sum_{i=1}^{4} A_{i}-\theta W \end{matrix}$$

b_i_- birth rate, contributes to the infant group only (b_1_>0, b_i_=0 for i=2,3,4)

*a_i_* – maturation rates from group i to group i+1

γ*_1_,* γ*_2_*- recovery rates for cholera cases and asymptomatic infections

ν- loss of natural immunity

*ξ_1_, ξ_2_* - excretion of *V. cholerae* by cholera cases and asymptomatically infected in water

*θ*- decay rate of *V. cholerae* in the environment

μ_i_- natural death rate by age group

*p* – proportion of the infections of non-vaccinated which become cholera cases

*q* – proportion of the infections of vaccinated which become symptomatically infected

N – total population size

N_i_ – population size by age group

*m* – migration rate

$d_{i}^{s}, d_{i}^{i}, d_{i}^{a}, d_{i}^{r}$- proportion of susceptibles, symptomatically infected, asymptomatically infected and recovered by age group among migrants who join the population. These proportions are calculated based on the monthly distribution of the population when simulated in absence of vaccination.

The forces of infections ($\lambda_{i}^{0} and \lambda_{i}^{j}$) represent the risk of the susceptible and vaccinated individuals in age group i to acquire cholera including short cycle transmission through contacts with infected individuals and long cycle transmission through exposure to contaminated water:

$$\begin{matrix} \lambda_{i}^{0}=(\sum_{k=1}^{4} I_{k}+\alpha\sum_{k=1}^{4} A_{k})\frac{s_{i}\beta_{H}}{N}+\frac{s_{i}\beta_{W}W}{k+W} \\ \lambda_{i}^{j}=(\sum_{k=1}^{4} I_{k}+\alpha\sum_{k=1}^{4} A_{k})\frac{(1-E^{j})s_{i}\beta_{H}}{N}+\frac{(1-E^{j})s_{i}\beta_{W}W}{k+W} \\ \beta_{W}=\left\{\begin{matrix} \beta_{W}^{\min}(1+\delta_{1}) rem(t,360)<60 \\ \beta_{W}^{\min}(1+\delta_{2}) \delta_{3}+150<rem(t,360)<\delta_{3}+210 \\ \beta_{W}^{\min} otherwise \end{matrix}\right. \end{matrix}$$

Where:

*β_H_* – transmission rate from cholera cases to unvaccinated susceptible adults by direct contact (human-to-human)

α – relative infectiousness of the asymptomatically infected.


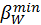
 - transmission rate to susceptibles adults due to the environment (water-to-human) outside of cholera season

δ_1_ (δ_2_) – seasonal multiplier for increased environmental risk of transmission during the spring and fall cholera seasons

δ_3_ – shift in the start of the fall cholera season

*β_W_* – transmission rate to susceptible adults from the environment (water-to-human)

s_i_ (i=1..4) – relative susceptibility of the group i. Adult group is used as a baseline (s_4_=1)

E^j^ – vaccine efficacy in reducing susceptibility when vaccinated at age 1-4 y (j=1), 5-14 y (j=1), 15+ y (j=3)

**Table 1. Mathematical model parameters for cholera transmission in Matlab, Bangladesh.** Values taken from Dimitrov et al 2014.

| Parameter | Description | Values and ranges |
| --- | --- | --- |
| *γ_1_ =γ_2_* | Recovery rates for cholera cases and asymptomatic infections (infectious time) | 5 days |
| *γ_3_* | Loss (daily) of natural immunity (protected time) | 3 years |
| b_1_ | Birth rate (annual) | 0.025 |
| *N_0_* | Initial population size | 220,000 |
| μ_i_ | Death rates (annual) by age groups (0-1, 2-4, 5-14, 15+) | 2%, 0.2%, 0.1%, 1% |
| k | Half-saturation concentration of cholera in the environment (water) | 10^5^ |
| *ξ_1_, ξ_2_* | Excretion rate (daily) of cholera by cholera cases [asymptomatically infected] in water | 100 [1] |
| *θ* | Death rate of cholera in water (survival time) | 30 days |
| p | Proportion of the infections of non-vaccinated individuals that become (symptomatic) cholera cases | 20% |
| q | Proportion of the infections of vaccinated individuals that become cholera cases | 20% |
| E^j^ | Vaccine efficacy in reducing susceptibility by age at the time of vaccination | 42% (1-4 years)  68% (5-14 years)  74% (15+ years) |
| *β_H_* | Transmission rates from direct contacts with cholera cases | 0.4477 |
| *β_asymp_* | Relative infectiousness of the asymptomatically infected | 0.1454 |
| 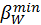 | Transmission rate from environment outside Cholera seasons | 2.258 x10^-6^ |
| δ_1_ (δ_2_) | Multipliers for increased transmission risk due to environment during Cholera seasons | 10-80 (spring)  15-150 (fall) |
| δ_3_ | Shift in the start of the fall Cholera season (days) | 0-60 |
| s_i_ | Relative susceptibility to Cholera by age group using adults (15+ years) as reference | 6.274 (0-1 years) 5.253 (2-4 years) 1.845 (5-14 years) |
| r | Proportion cholera cases reported | 10% |
| InitR | Fraction of the population initially in the recovered compartment | 18.1% |

## 2. Modeling cholera vaccination strategies

We modeled the annual vaccination campaigns reaching 70% of one-year-olds combined with vaccination campaigns targeting young children, all children, or the entire population every 3 years. Those younger than one year were excluded from vaccination. Vaccination of one-year-olds in all scenarios is modeled by targeting half of the population younger than 2 years old. Vaccination campaigns are assumed to achieve 70% coverage among children and 55% among adults.

Each vaccination campaign is modeled by instantaneous transfer of susceptible (S) and recovered (R) compartments into vaccinated compartment (V)at regular 1-year or 3-year intervals. Therefore , the number of vaccinations per a campaign with X% coverage in age group *i* is X% of (S_i_+R_i_ +V_i_ ).

Simulations without vaccinations are initiated with no vaccinated ($V_{i}^{j}$=0) and no further transfers to the vaccinated compartments. We conservatively assume that vaccine administered during campaigns protects for exactly three years, after which vaccinated individuals return to full susceptibility. Vaccine coverage under this assumption is depicted in Figure S2.

A) B)


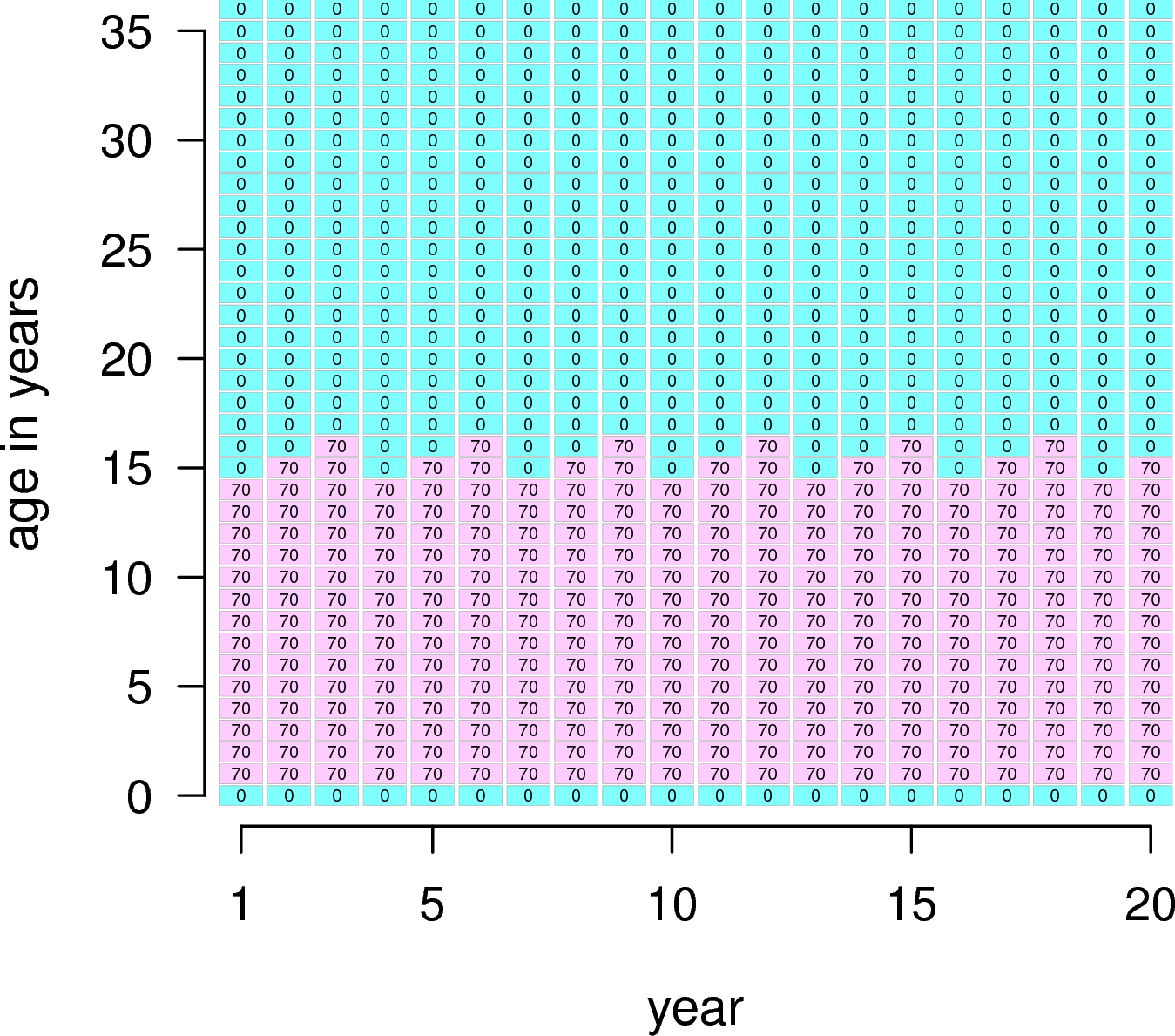

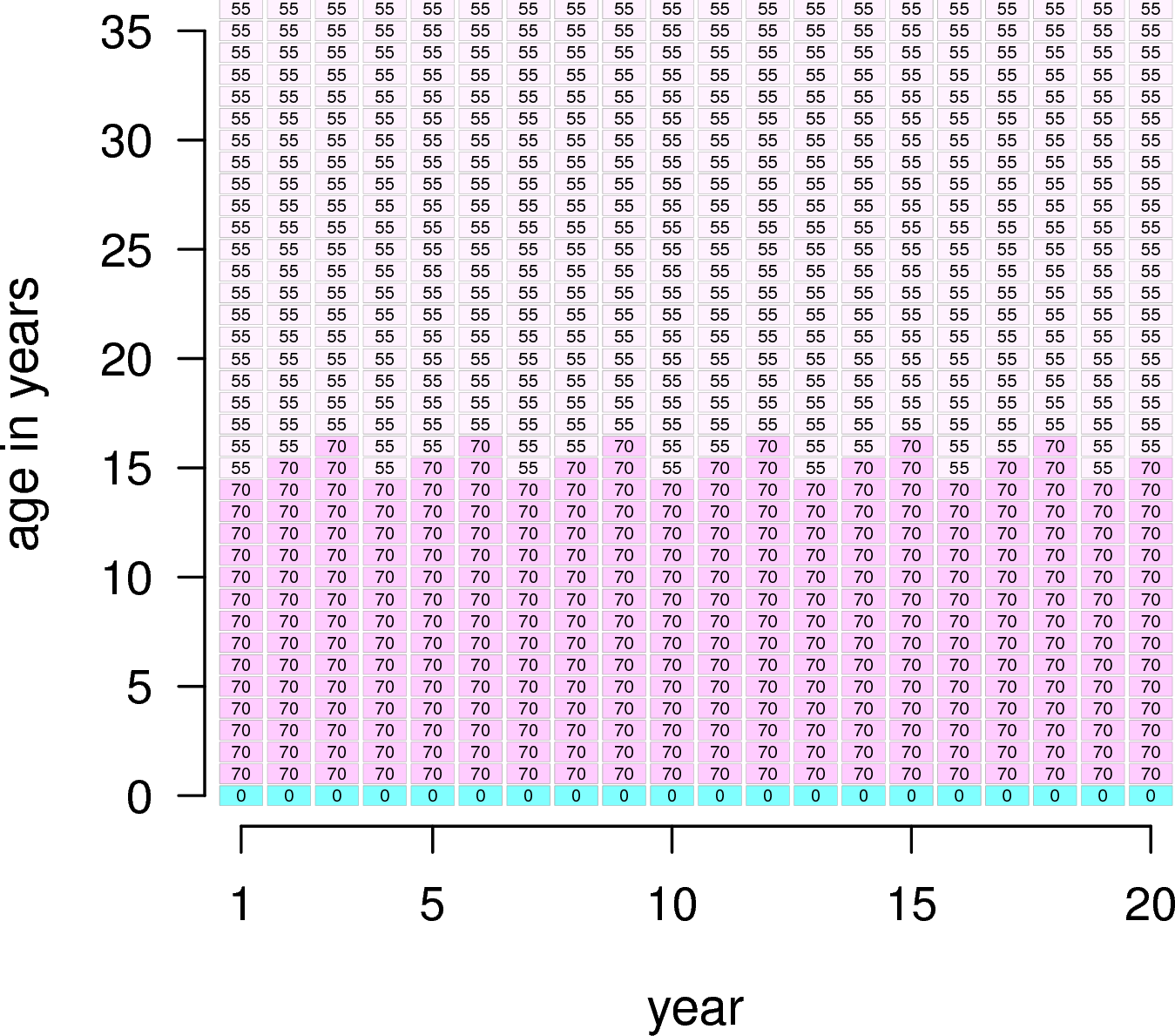


C) D)


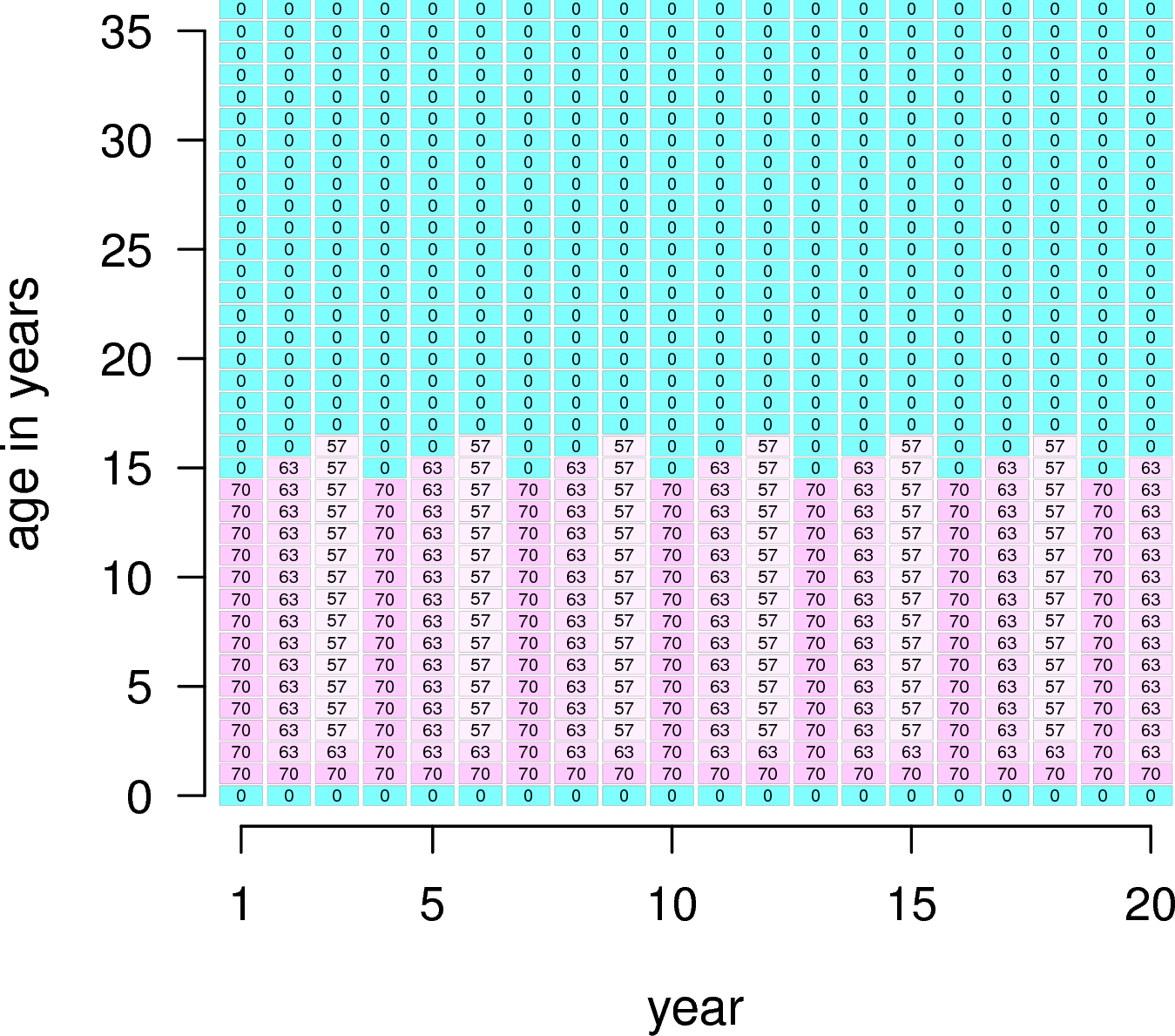

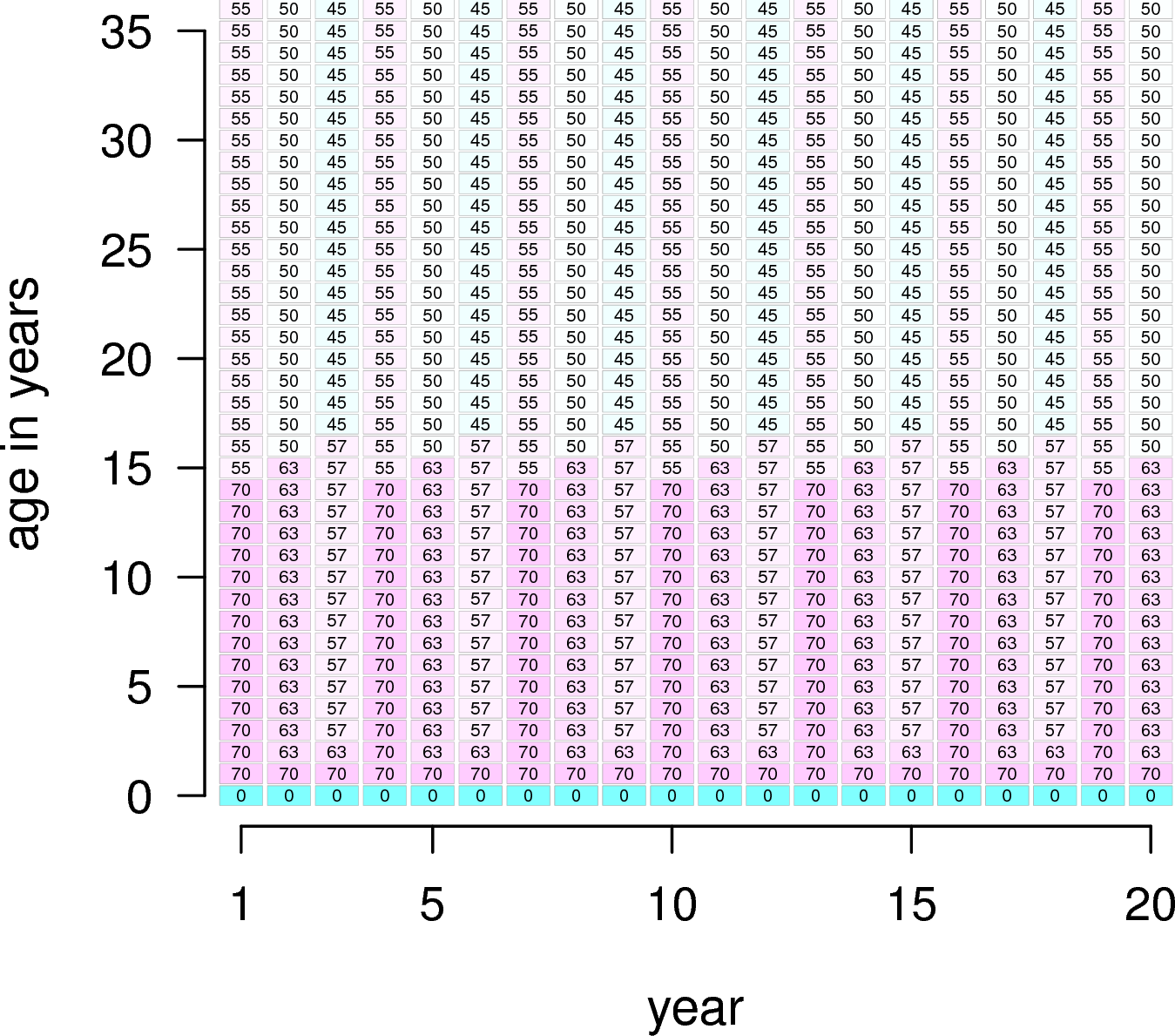


E) F)


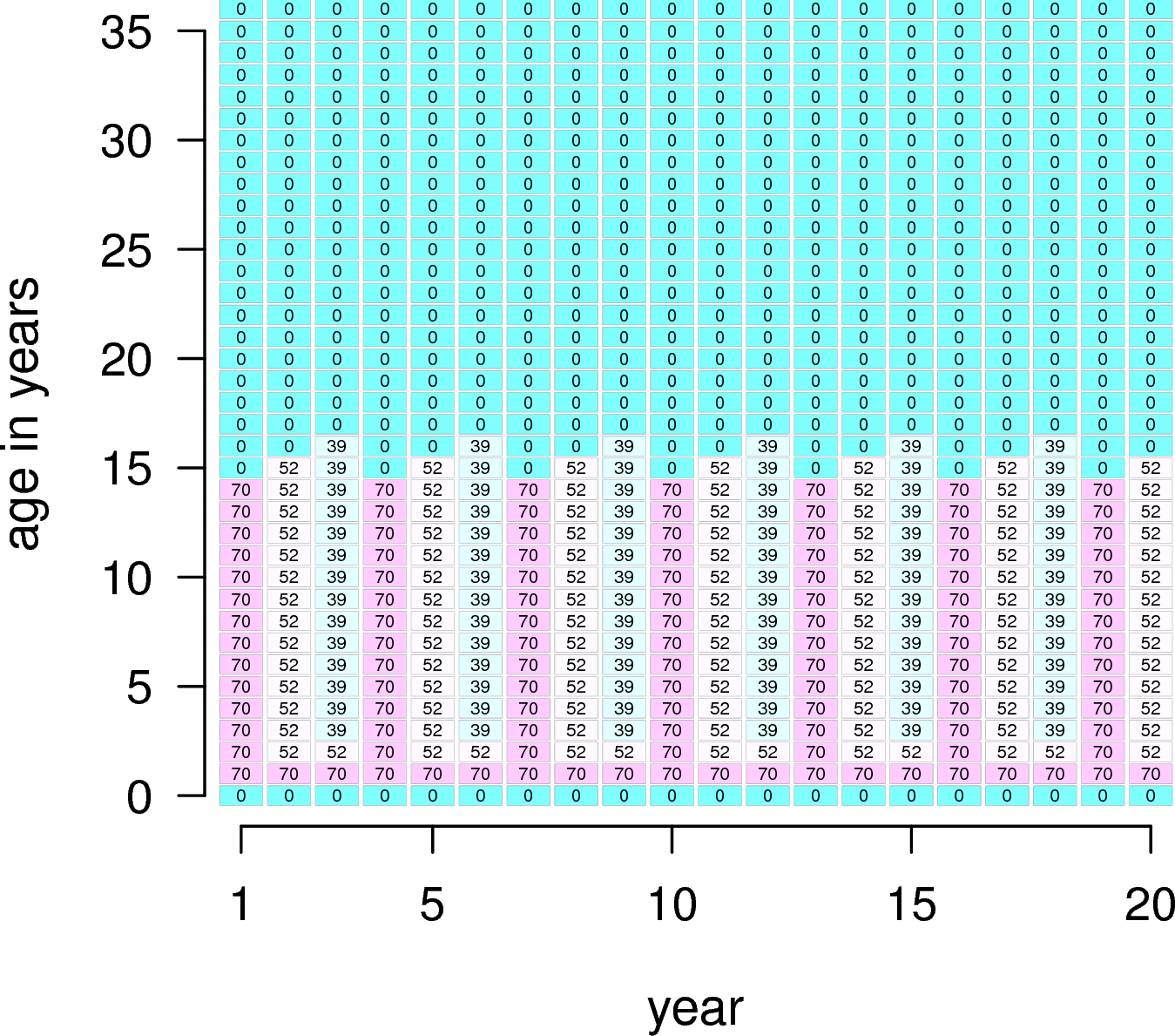

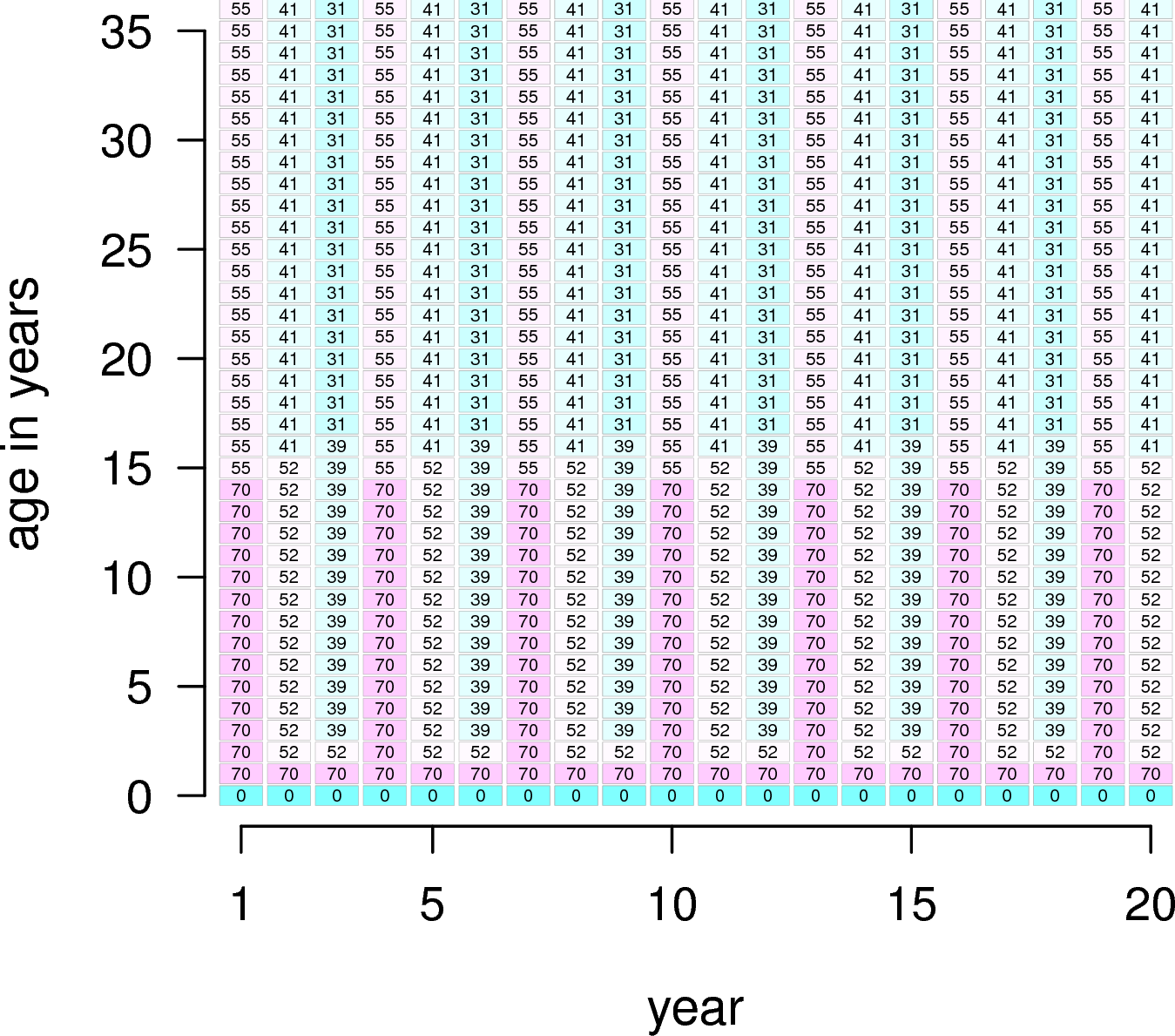


**Figure S2. Diagram of coverage and migration rates.** These diagrams show the approximate OCV coverage by one-year age cohorts. Dark pink indicates high coverage for an age group, while dark blue indicates low coverage. Panels A, C, and E show 3-year campaigns that cover only those from ages 1 to under 15 years (70% coverage), and panels B, D, and F show those that cover the whole population 1 year old and older (70% coverage for those under 15, 55% otherwise). Scenarios with 0% (panels A and B), 10% (C and D), and 25% (E and F) annual migration were simulated. We do not show the campaigns that cover ages 1 to 4. There is annual “routine” vaccination of 1-year-olds.

## 3. Translating model results to a population in Dhaka

The model was calibrated to simulate the dynamics of cholera in Matlab. Below, we outline the procedure we used to convert the number of symptomatic cases of cholera in our Matlab model to an estimate of the number of reported cases in Dhaka, which has a different reporting rate and age structure. We assume that the size of the at-risk population in Dhaka is fixed over time.

We computed the number of symptomatic cholera infections per age group in Matlab and translated these to annual incidence of cholera by age group. The age structure in the model was based on Matlab, Bangladesh. Using the population projection for 2015 published with the 2011 census data, we estimated the fraction of the population of Bangladesh in each age group. We translated the model results to the age structure of the Bangladesh by multiplying the annual symptomatic incidence in each age group in the model by the 2015 Bangladesh population proportions. Using these figures, we assume that the Dhaka population was 4.2% 0-1 year olds, 5.6% 2-4 year olds, 22.1% 5-14 year olds, 60.2% 15-59 year olds, and 7.9% 60 years and older. We assumed that the population size was fixed over the 10 years of simulation.

We had to compute a reporting rate to translate the incidence of symptomatic cholera in the model for one year to the Mirpur estimate of 2.3 reported cases per 1000 population. We computed this by dividing the average annual number of symptomatic infections from 100 stochastic runs of the “no vaccination” scenario by 2.3. Thus, we have estimates of the number of cases of cholera in Dhaka under different vaccination scenarios normalized to an incidence of 2.3 reported cases per 1000 population per year. With population-level reported incidence normalized to 2.3 per 1,000 population, we find an incidence of 7.86 per 1,000 among those under 5 years old, 2.65 per 1,000 among those from ages 5 years to under 15, and 1.38 per 1000 among those 15 years old and older.

**4. Supplemental modeling results**

**A) B)**

**
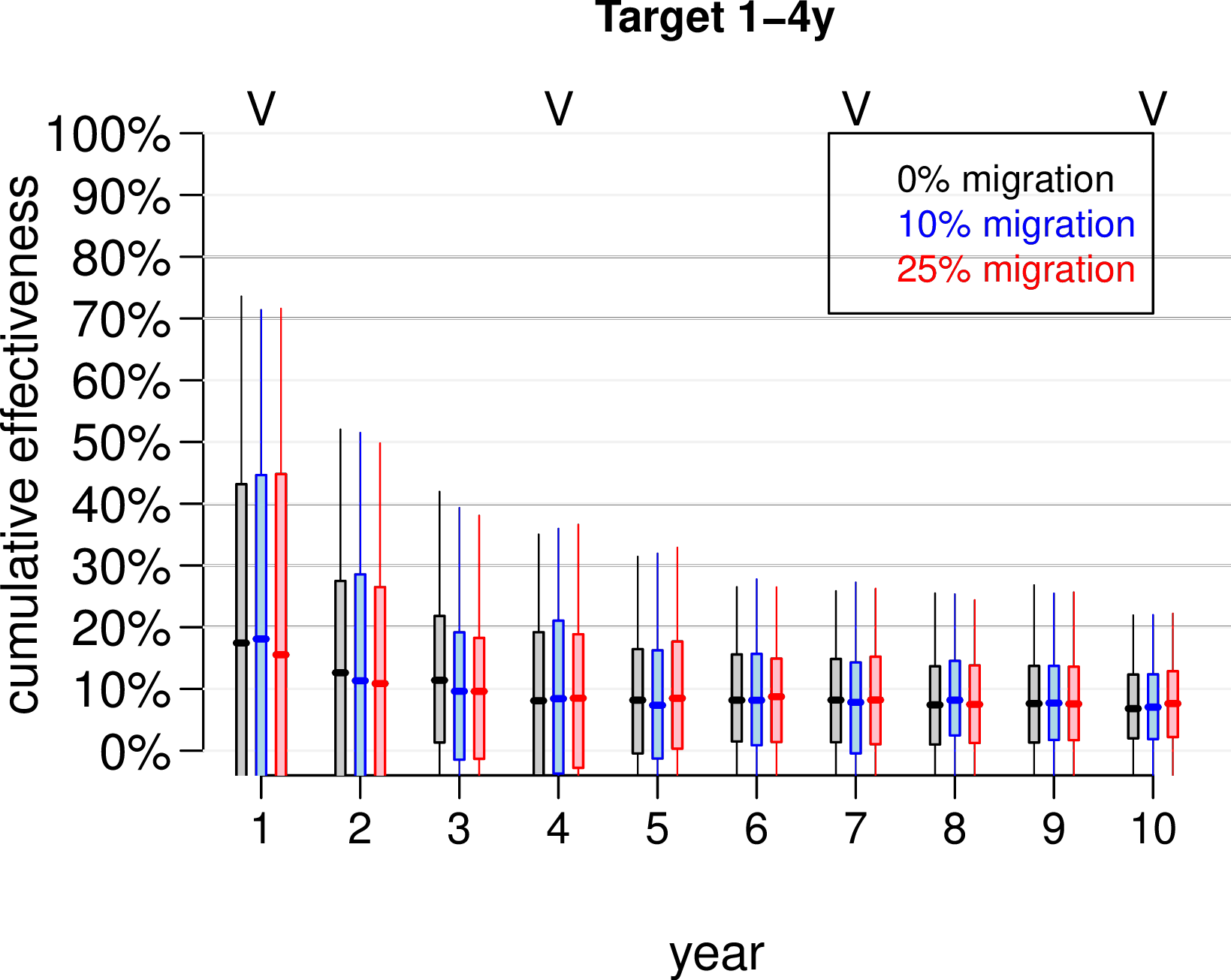

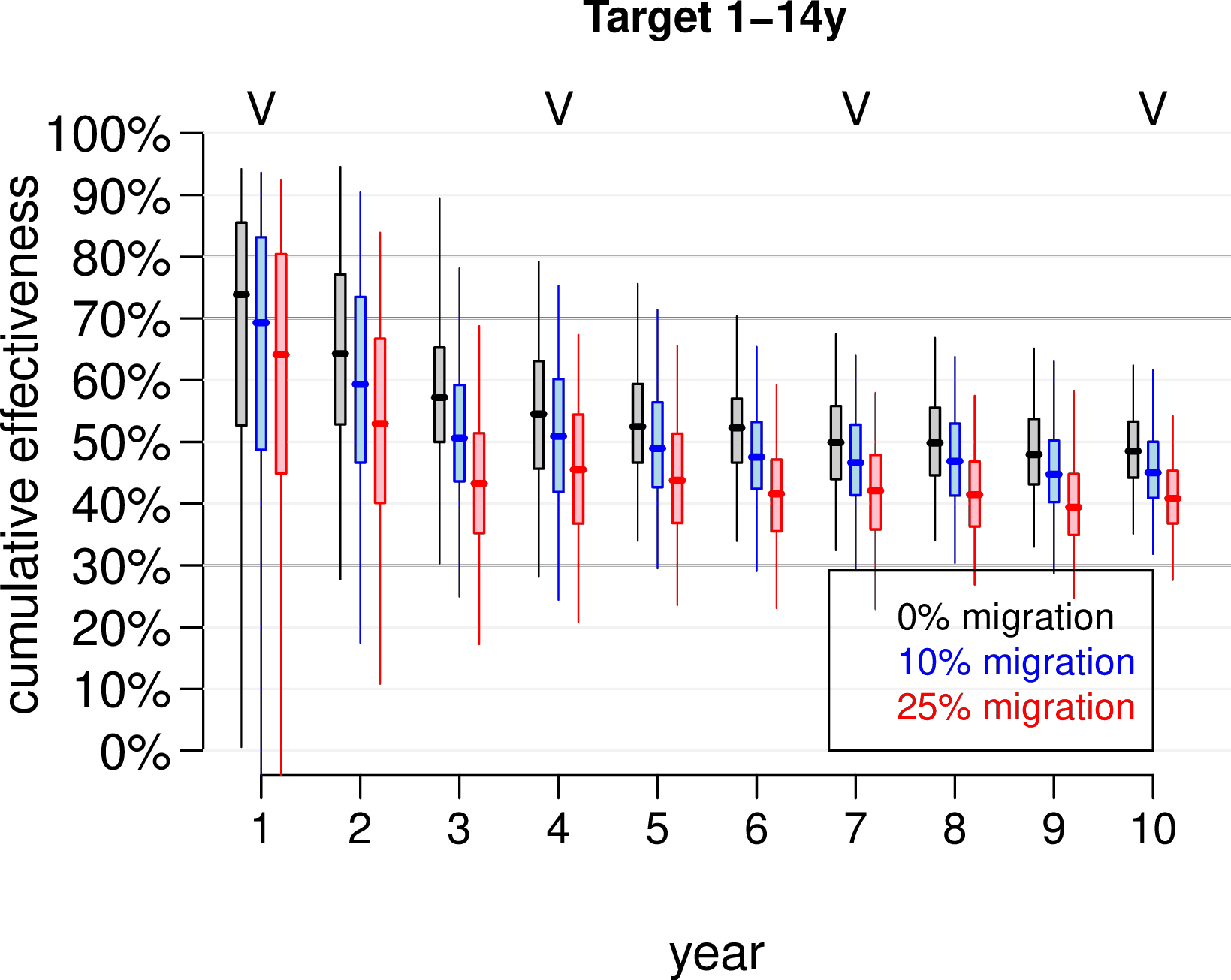
**

**C)**

**
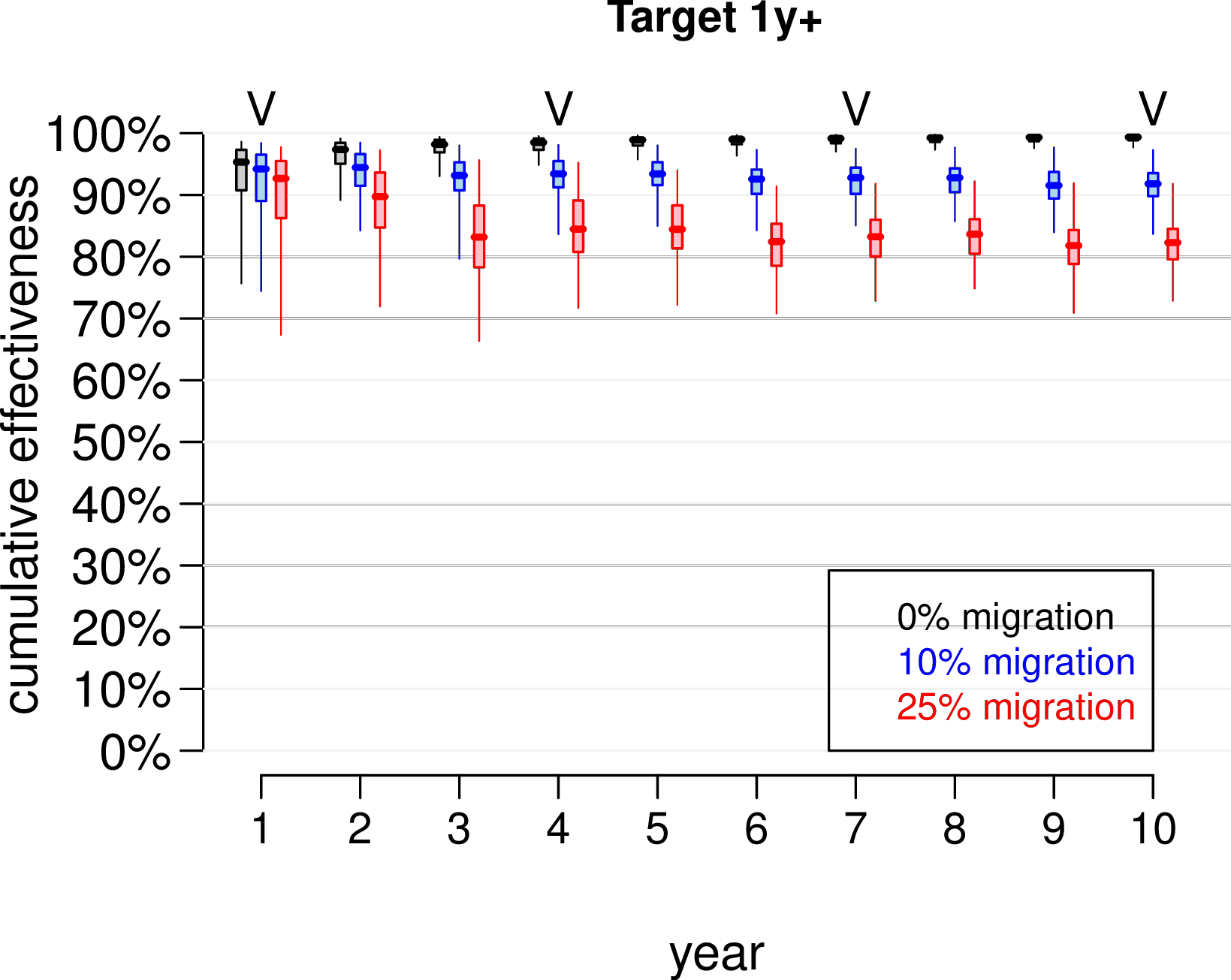
**

**Figure S3. Simulated cumulative effectiveness of vaccination.** The overall cumulative effectiveness (cumulative reduction of cases) of annual vaccination of 70% of 1-year-olds accompanied by mass vaccination of different age targets every 3 years: A) targeting those from 1 to under 5 years old, B) targeting those from 1 to under 15 years old, and C) targeting those ages 1 year and older. The model was run 100 times for each scenario, and bootstrap estimates of effectiveness were computed by comparing the incidences of 1000 pairs drawn with replacement from the vaccinated and unvaccinated (baseline) scenarios. The rectangles indicate the middle two quartiles of estimates, the horizontal line the median estimate, and the whiskers cover 95% of estimates.

**A) B)**

**
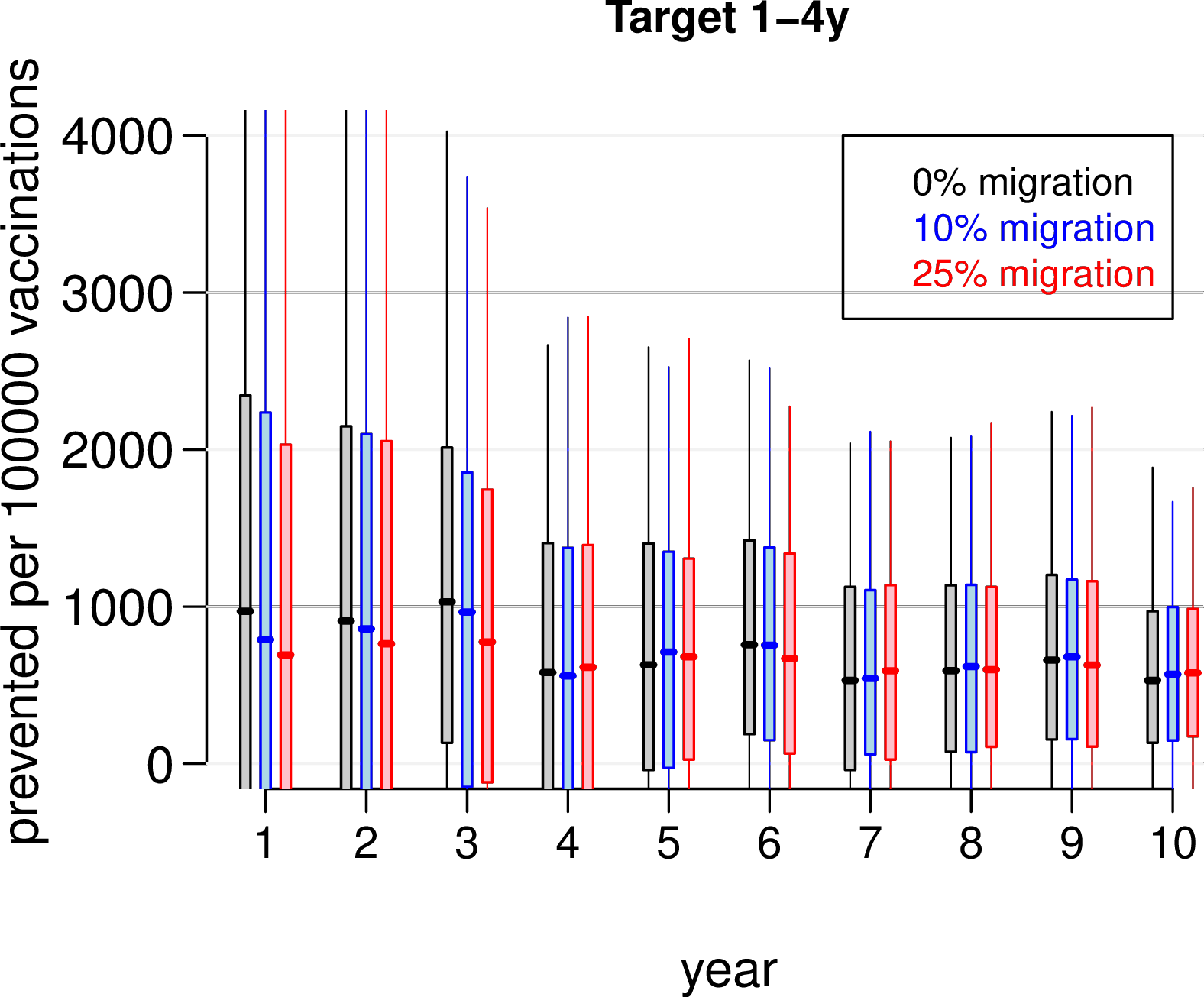

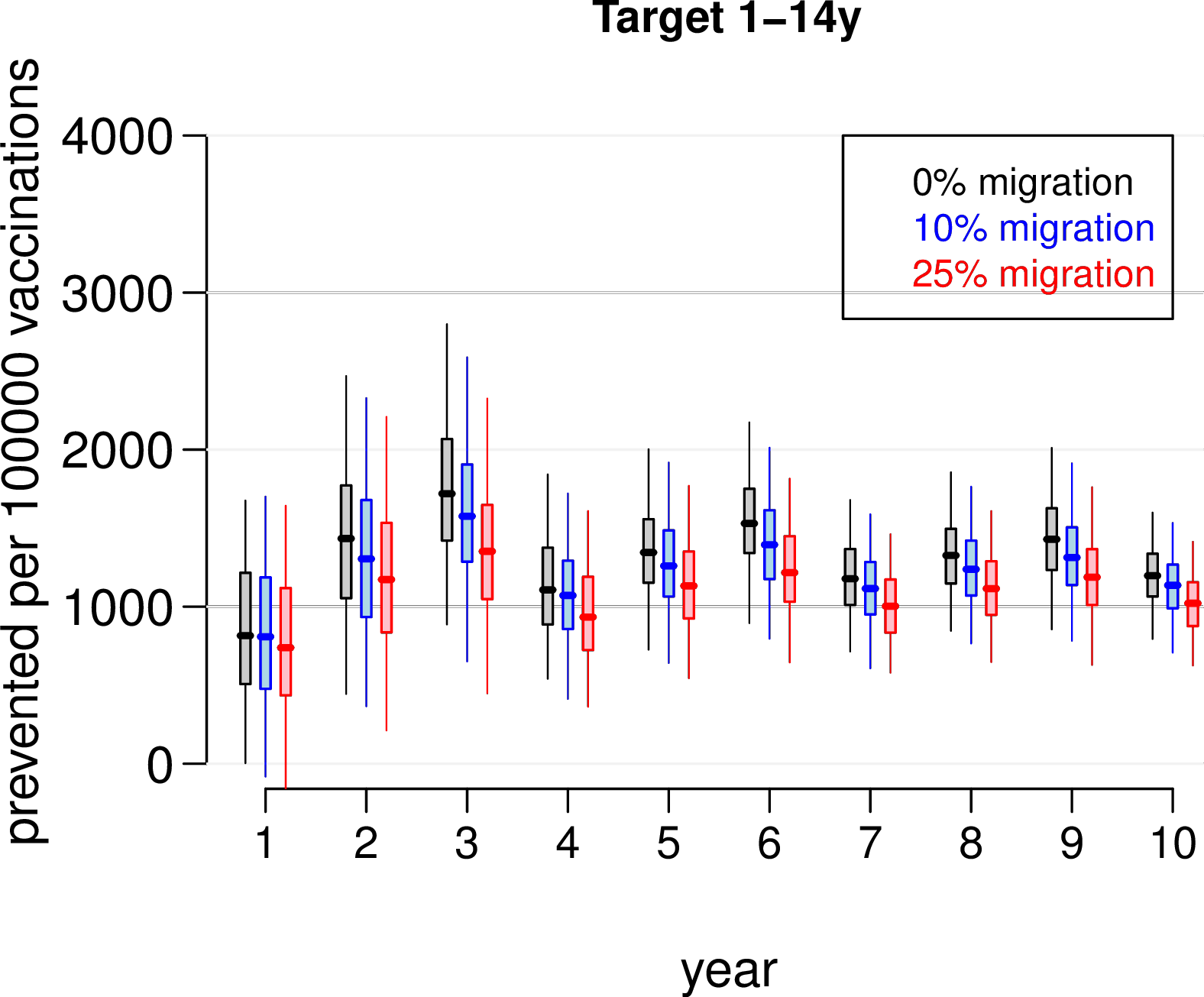
**

**C)**


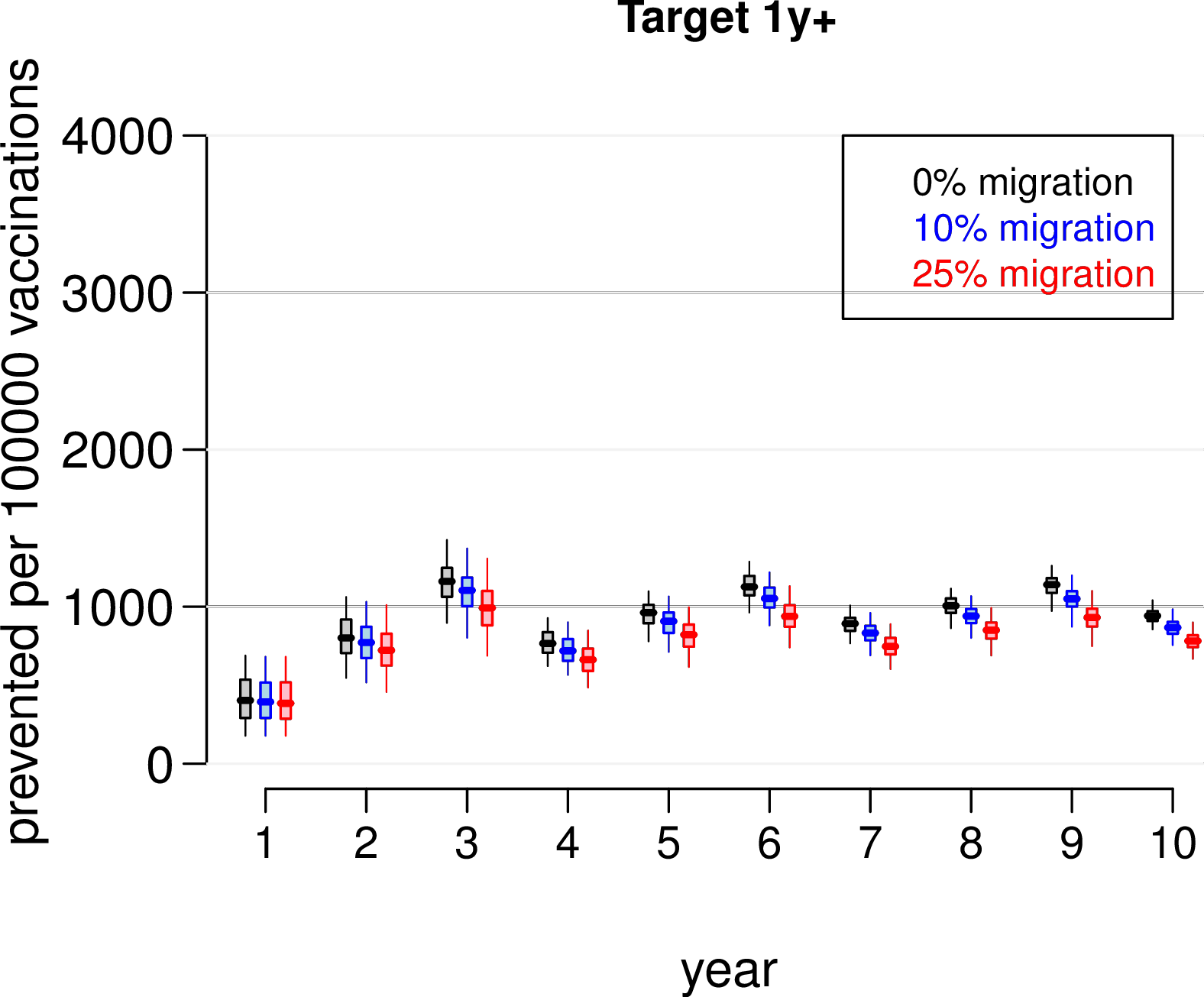


**Figure S4. Simulated cumulative reported cases prevented per 100,000 people vaccinated.** The overall efficiency (prevented reported cases per 100,000 vaccines used) of annual vaccination of 70% of one-year-olds accompanied by mass vaccination of different age targets every 3 years: A) targeting those from 1 to under 5 years old, B) targeting those from 1 to under 15 years old, and C) targeting those ages 1 year and older. Migration could either replace 0%, 10%, or 25% of the population each year. The model was run 100 times for each scenario, and bootstrap estimates of efficiency were computed by comparing the incidences of 1000 pairs drawn from the vaccinated and unvaccinated (baseline) scenarios with replacement. The rectangles indicate the middle two quartiles of estimates, the horizontal line the median estimate, and the whiskers cover 95% of estimates.

## 4. Vaccination campaigns every 5 years instead of 3

We simulated vaccination campaigns every 5 years instead of every 3 years, accompanied by annual vaccination of 1-year-olds. We assume that vaccine protects for 5 years in this scenario, which may be optimistic for young children. For the first 3 years after a campaign, effectiveness is the same as simulations when campaigns are every 3 years (compare Figure 3A from the main manuscript with Figure S5A below). In years 4 and 5, the number of cases grows, particularly when ages 1-14y or 1+y are targeted by campaigns. The average reduction in cholera incidence over 10 years is somewhat lower than when campaigns are every 3 years (compare Figure S5B with Figure 4: 41% vs 45% when ages 1-14 are targeted and 86% vs 91% when ages 1+ are targeted). This difference becomes larger when migration is 25% instead of 10% (compare Figure S5C with Figure S5D: 33% vs 41% when ages 1-14 are targeted and 68% vs 82% when ages 1+ are targeted).

A) B)


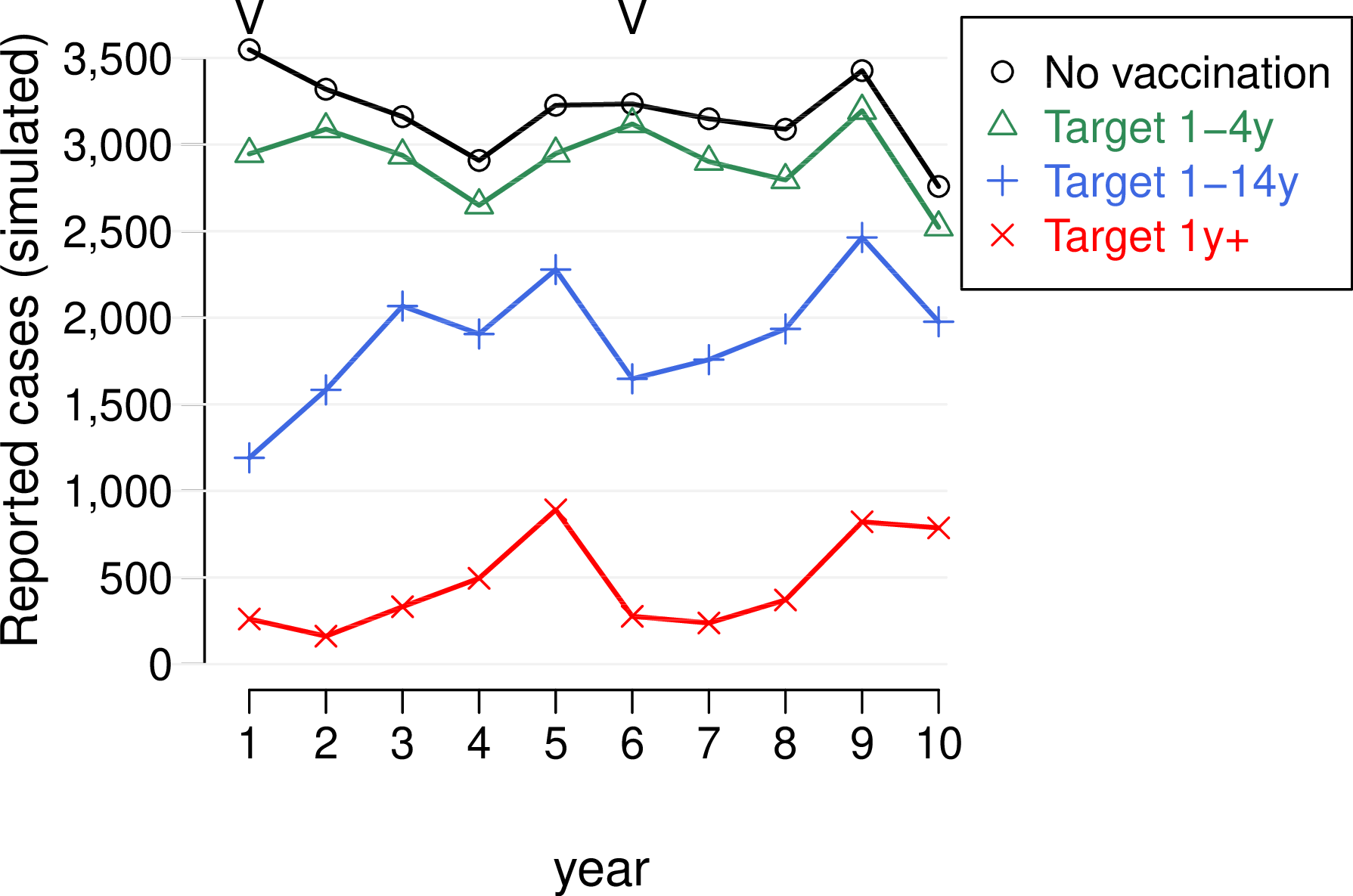

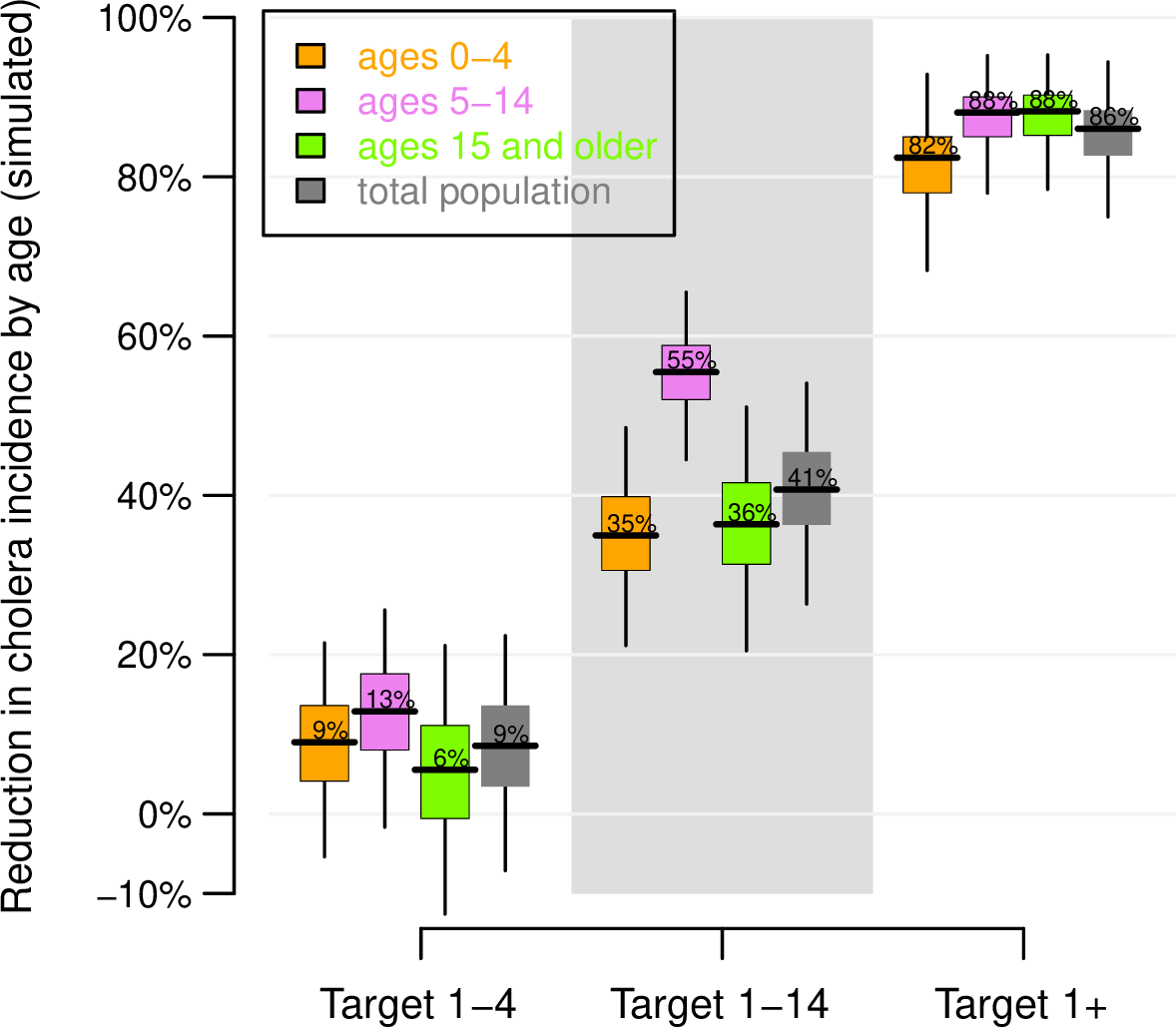


C) D)


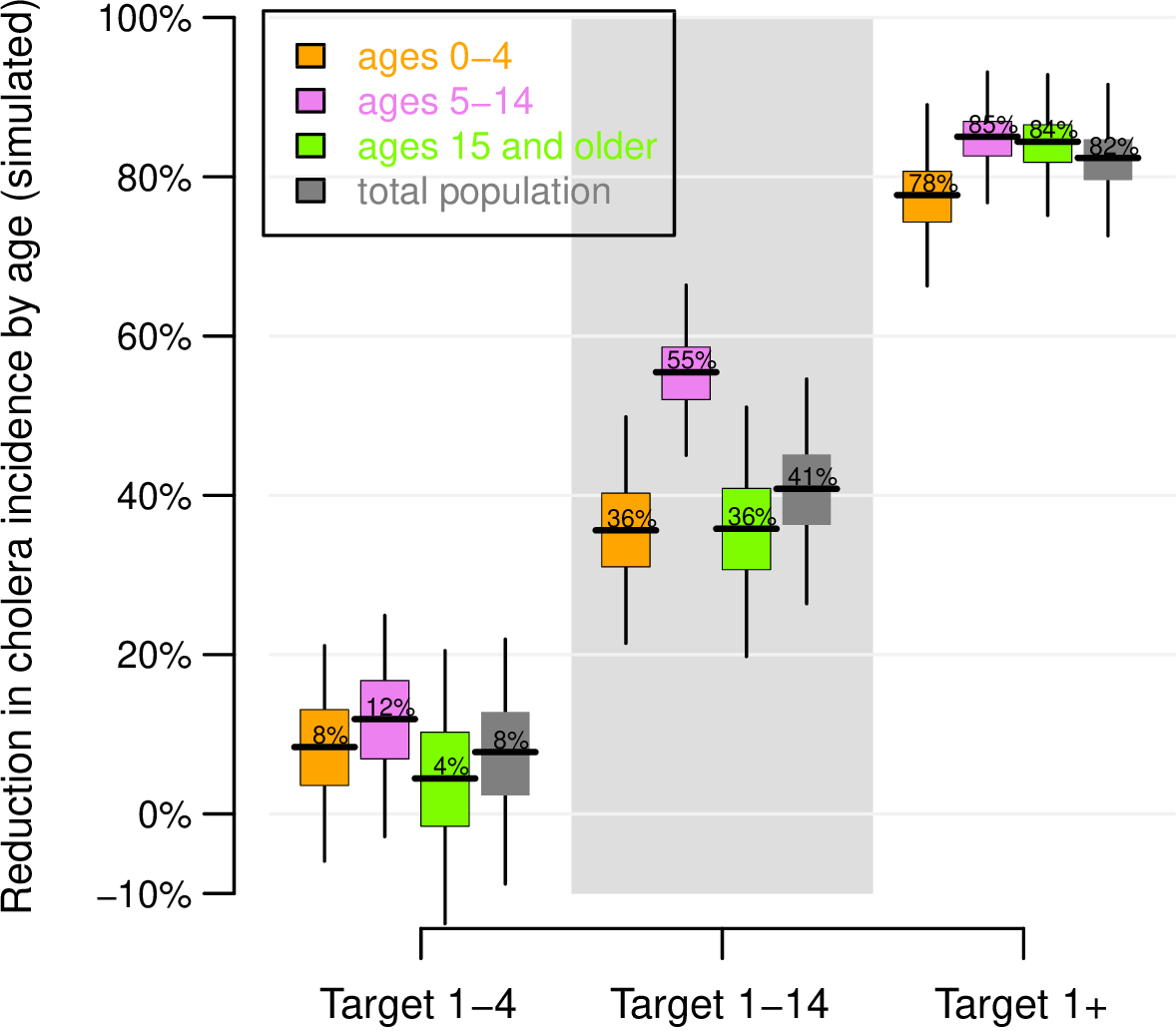

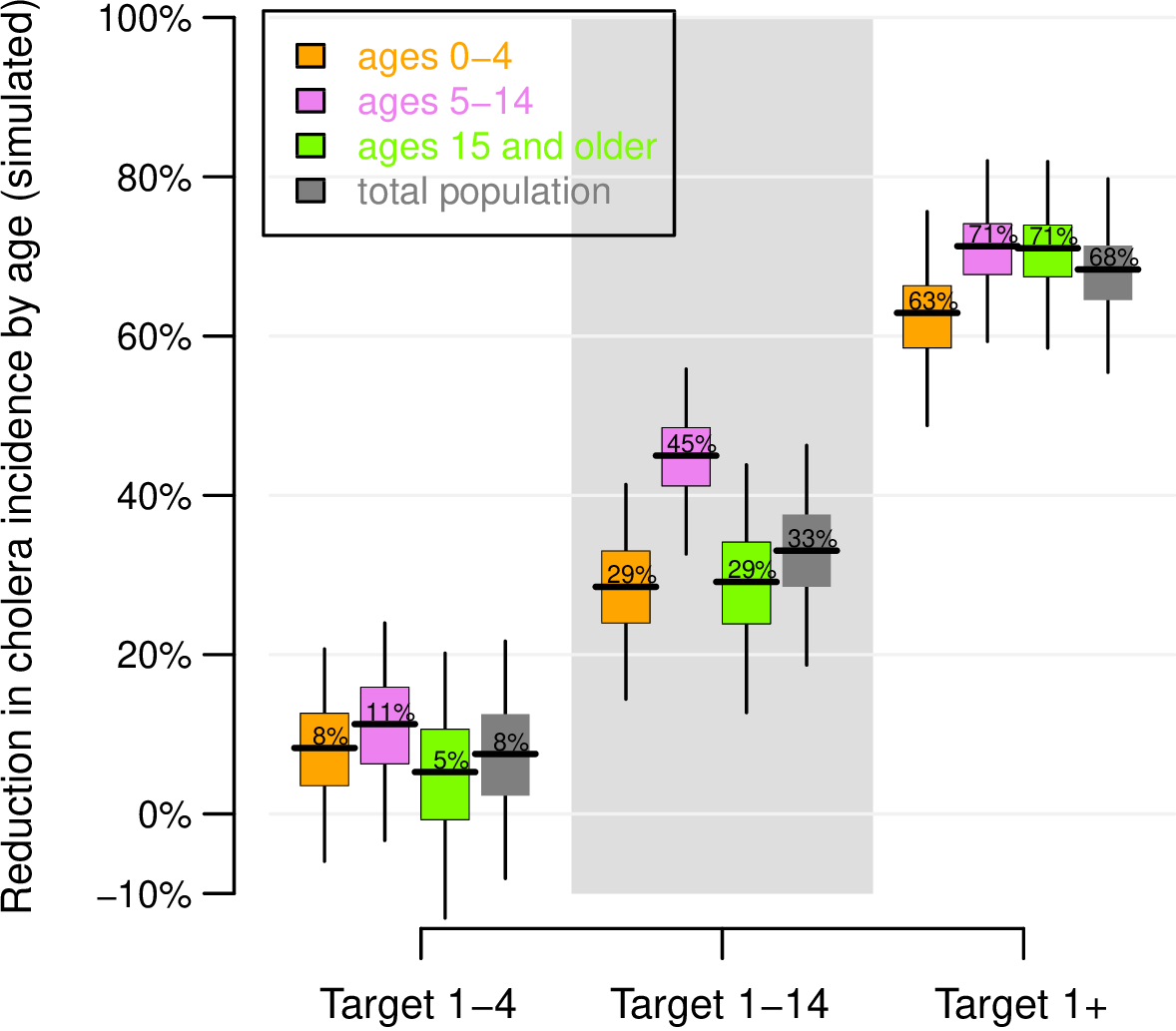
**Figure S5. Simulations of 5-year vaccination campaigns.** A) The simulated number of reported cases. Different target populations were targeted for vaccination in the model, and the average of 100 stochastic runs per strategy is plotted. The Vs above the lines indicate the years when vaccination campaigns occurred. B) The percent reduction in cholera incidence by age group over 10 years with respect to simulations with no vaccination are plotted. The distribution of effectiveness estimates is computed by taking 10,000 random draws with replacement from 100 stochastic runs with vaccination and 100 stochastic runs with no vaccination. The boxes indicate the inter-quartile interval of 100 model runs, the horizontal lines the median result, and the vertical lines the 95% observed interval. C) Reduction in cholera incidence when annual migration is 25% and vaccination campaigns are every 5 years. Results in the main text assume 10% annual migration. D) Reduction in cholera incidence when annual migration is 25% and vaccination campaigns are every 3 years.

## References

Bhattacharya SK, Sur D, Ali M, Kanungo S, You YA, Manna B, Sah B, Niyogi SK, Park JK, Sarkar B, Puri MK, Kim DR, Deen JL, Holmgren J, Carbis R, Dhingra MS, Donner A, Nair GB, Lopez AL, Wierzba TF, Clemens JD. [5 year efficacy of a bivalent killed whole-cell oral cholera vaccine in Kolkata, India: a cluster-randomised, double-blind, placebo-controlled trial.](http://www.ncbi.nlm.nih.gov/pubmed/24140390) Lancet Infect Dis. 2013 Dec;13(12):1050-6. doi: 10.1016/S1473-3099(13)70273-1.

Dimitrov DT, Troeger C, [Halloran ME](http://www.cidid.org/betz-halloran/), [Longini IM Jr.](https://en.wikipedia.org/wiki/Ira_Longini), Chao DL. Comparative effectiveness of different strategies of oral cholera vaccination in Bangladesh: A modeling study. [PLoS Negl Trop Dis](http://journals.plos.org/plosntds/). 8(12):[e3343](http://www.plosntds.org/article/info:doi/10.1371/journal.pntd.0003343). 2014.
